# The conserved Trp114 residue of thioredoxin reductase 1 has a redox sensor-like function triggering oligomerization and crosslinking upon oxidative stress related to cell death

**DOI:** 10.1038/cddis.2014.574

**Published:** 2015-01-22

**Authors:** J Xu, S E Eriksson, M Cebula, T Sandalova, E Hedström, I Pader, Q Cheng, C R Myers, W E Antholine, P Nagy, U Hellman, G Selivanova, Y Lindqvist, E S J Arnér

**Affiliations:** 1 Division of Biochemistry, Department of Medical Biochemistry and Biophysics, Karolinska Institutet, SE-17177 Stockholm, Sweden; 2 Division of Molecular Structural Biology, Department of Medical Biochemistry and Biophysics, Karolinska Institutet, SE-17177 Stockholm, Sweden; 3 Department of Microbiology, Tumor and Cell Biology, Karolinska Institutet, SE-17177 Stockholm, Sweden; 4 Department of Pharmacology and Toxicology, Medical College of Wisconsin, 8701 Watertown Plank Road, Milwaukee, WI 53226, USA; 5 Department of Biophysics, Medical College of Wisconsin, 8701 Watertown Plank Road, Milwaukee, WI 53226, USA; 6 Department of Molecular Immunology and Toxicology, National Institute of Oncology, Rath György ut 7-91, 1122, Budapest, Hungary; 7 Ludwig Institutet for Cancer Research Ltd., Uppsala University BMC, SE-75 124 Uppsala, Sweden

## Abstract

The selenoprotein thioredoxin reductase 1 (TrxR1) has several key roles in cellular redox systems and reductive pathways. Here we discovered that an evolutionarily conserved and surface-exposed tryptophan residue of the enzyme (Trp114) is excessively reactive to oxidation and exerts regulatory functions. The results indicate that it serves as an electron relay communicating with the FAD moiety of the enzyme, and, when oxidized, it facilitates oligomerization of TrxR1 into tetramers and higher multimers of dimers. A covalent link can also be formed between two oxidized Trp114 residues of two subunits from two separate TrxR1 dimers, as found both in cell extracts and in a crystal structure of tetrameric TrxR1. Formation of covalently linked TrxR1 subunits became exaggerated in cells on treatment with the pro-oxidant p53-reactivating anticancer compound RITA, in direct correlation with triggering of a cell death that could be prevented by antioxidant treatment. These results collectively suggest that Trp114 of TrxR1 serves a function reminiscent of an irreversible sensor for excessive oxidation, thereby presenting a previously unrecognized level of regulation of TrxR1 function in relation to cellular redox state and cell death induction.

The selenoprotein thioredoxin reductase 1 (TrxR1) reduces the active site of thioredoxin 1 (Trx1) using NADPH and is thereby required for the many actions of reduced Trx1, including the promotion of cell viability and proliferation^1, 2, 3^ or the regulation of signaling pathways through modulation of receptor-linked protein phosphorylation cascades.^2, 3, 4, 5^ Such signaling is intrinsically complex and involves compartmentalized NADPH oxidases, producing bursts of superoxide and/or hydrogen peroxide that transiently inhibit protein tyrosine phosphatases, possibly through peroxiredoxin intermediates.^2, 3, 6^ Hydrogen peroxide-driven signaling would be facilitated if the reducing Trx system could be inhibited, at least in a compartmentalized manner, by an oxidative burst. Such physiological inhibition might at first seem counterintuitive, since the Trx system is known as a major reductive enzyme system providing protection against oxidation. Early findings nonetheless showed that TrxR1 can be specifically inhibited on receptor-linked signaling, as exemplified with EGF treatment of cells where oxidation of the TrxR1 selenocysteine residue was the suggested inhibitory molecular mechanism.^7^ Other observations suggest alternative mechanisms for redox sensing through TrxR1. The major form of TrxR1 is a non-covalently linked homodimer,^8, 9^ whereas higher oligomers including tetramers have also been described.^10, 11^ Interestingly, an ≈110 kDa TrxR1-positive immunoreactive band is observed in reducing SDS-PAGE analyses of protein lysates obtained from cancer cells treated with the p53-reactivating compound RITA (NSC652287).^12^ Formation of those TrxR1 protein species (originally reported as ≈130 kDa) directly correlates with RITA-triggered cell death^13^ and excessive cellular oxidative stress.^14, 15^ The size of that protein band was surprising because there is no known TrxR1-encoding transcript encoding for a polypeptide of such large size; the subunit size of the main TrxR1 form is ≈55 kDa,^16^ thereby indicating that a band migrating in a denaturing reducing gel as ≈110–130 kDa could possibly consist of covalently non-disulfide-linked pairs of subunits. Because regulated protein oligomerization is a general phenomenon in signaling,^17^ we here wished to further explore this new form of TrxR1. The results led us to the identification of the surface-exposed Trp114 residue of TrxR1 essentially serving the role of a redox sensor, as it is suggested to communicate with the FAD of TrxR1, become easily oxidized and affecting both enzymatic activity and oligomerization state of the enzyme in response to oxidation.

## Results and Discussion

**Figure 1:**
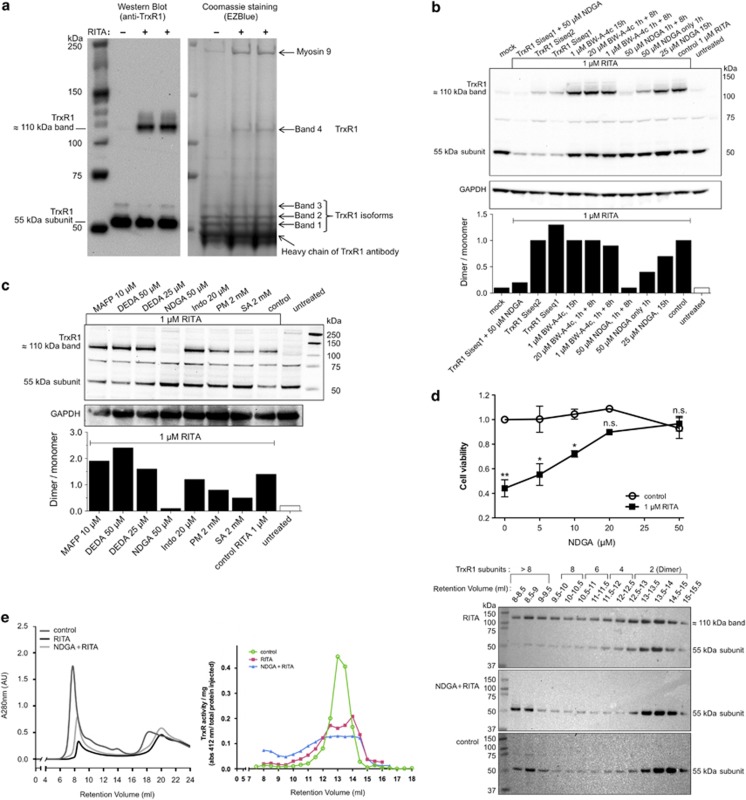
RITA treatment promotes the formation of covalently linked TrxR1 oligomers in cancer cells that can be prevented by NDGA and correlate with cell death. (**a**) RITA (1 *μ*M) treatment of HCT116 cells for 8 h triggers the formation of an ≈110 kDa TrxR1-positive band seen in western blots.^13^ Here TrxR1-derived protein species were enriched from HCT116 cells (controls or treated with RITA, as indicated) by immunoprecipitation, visualized by Coomassie staining and further analyzed by tryptic digests and mass spectrometry (Supplementary Table S1). (**b**, **c**) Pre-treatment of HCT116 cells using 50 *μ*M NDGA for 1 h blocked RITA-induced formation of TrxR1 dimeric bands as seen on western blots, also further verified using knockdown of TrxR1 with siRNA (Siseq1 and Siseq2). Below the western blots, the results of densitometric quantifications of dimer over monomer ratios are shown. See text for details. DEDA, 7,7-dimethyl-(5Z,8Z)-eicosadienoic acid 25 or 50 *μ*M (phospholipase A2 inhibitor, sPLA2 and cPLA2); Indo, indomethacin 20 *μ*M (Cox 1, 2 inhibitor); MAFP, methyl arachidonyl fluorophosphonate 10 *μ*M (phospholipase A2 inhibitor, cPLA2 and iPLA2); NDGA, nordihydroguareric acid 50 *μ*M; PM, pyridoxamine dihydrochloride 2 mM; SA, salicylamine 2 mM (to scavenge lipid-derived ketoaldehydes). (**d**) NDGA treatment prevents RITA-induced cell death in HCT116 cells. (**e**) TrxR1 oligomers in cell lysates, as indicated, were fractionated using gel filtration (left), whereupon all fractions were analyzed for TrxR activity (middle) and band sizes as detected using western blot of reducing SDS-PAGE analyses (right). The expected migration over the gel filtration column of different TrxR1 oligomers in solution is also indicated as the number of TrxR1 subunits that would be required to yield the corresponding elution (top, right panel)

Utilizing immunoprecipitation (Figure 1a) followed by mass spectrometric analyses (Supplementary Table S1), we first positively identified the ≈110 kDa TrxR1-immunoreactive band formed in RITA-treated HCT116 cells as indeed being TrxR1. Interestingly, this ≈110 kDa form of TrxR1 appeared to have a modified MIEAVQNHIGSLNWGYR peptide (covering residues 101–117, with numbering as in the common TXNRD1_v1 splice variant^16^). This 101–117 peptide was found in ≈55–65 kDa subunits of TrxR1 (bands 1–3, Figure 1a) as both unmodified and oxidized forms, but could not be found in tryptic digests of the ≈110 kDa protein (band 4; Supplementary Table S1). Assuming that oxidation may have a role in formation of the ≈110 kDa TrxR1 species, we next analyzed whether antioxidant treatment could prevent its formation. Indeed, treatment with nordihydroguaiaretic acid (NDGA), a highly potent antioxidant,^18^ prevented formation of the ≈110 kDa band on RITA treatment (Figure 1b). Because NDGA can also be a lipoxygenase inhibitor, we tested the specific lipoxygenase inhibitor BW-A-4c, but this had no effect on the ability of RITA to induce the ≈110 kDa band (Figure 1b). Similarly, the phospholipase A_2_ inhibitors methyl arachidonyl fluorophosphonate (MAFP) or 7,7-dimethyl-(5Z,8Z)-eicosadienoic acid (DEDA) or the cyxlooxygenase inhibitor indomethacin, did not prevent formation of the ≈110 kDa band, whereas the nonspecific lipid-derived ketoaldehyde scavengers pyridoxamine or salicylamine partially decreased the ≈110 kDa species but not nearly as much as NDGA (Figure 1c). NDGA furthermore protected cells from RITA-induced cell death (Figure 1d). Using gel filtration analyses of crude protein lysates, extracted from treated cells, we found that RITA promoted the formation of modified species of TrxR1 that contained higher proportions of the ≈110 kDa band and migrated in gel filtration as both dimeric and higher multimeric variants. Formation of the ≈110 kDa band upon RITA treatment, as well as a higher proportion of larger multimeric forms of TrxR1, was counteracted by NDGA (Figure 1e). Trace amounts of higher TrxR1 oligomers (i.e., with migration in gel filtration corresponding to more than two 55 kDa subunits) were, however, seen in both control cells and NDGA+RITA-treated cells (Figure 1e, retention volumes <12 ml, western blots). TrxR activity was mainly found in the fractions containing dimeric enzyme (retention volume 12–15 ml, Figure 1e, middle) while a low activity was also detected in the fractions containing higher oligomers (retention volumes <12 ml, Figure 1e, middle). Collectively these findings suggested that RITA-triggered modifications of TrxR1 promoted the formation of oligomeric TrxR1 complexes, containing oxidative modifications of the peptide spanning amino acid positions 101–117 and, also, that such events correlated with RITA-triggered induction of cell death.

**Figure 2:**
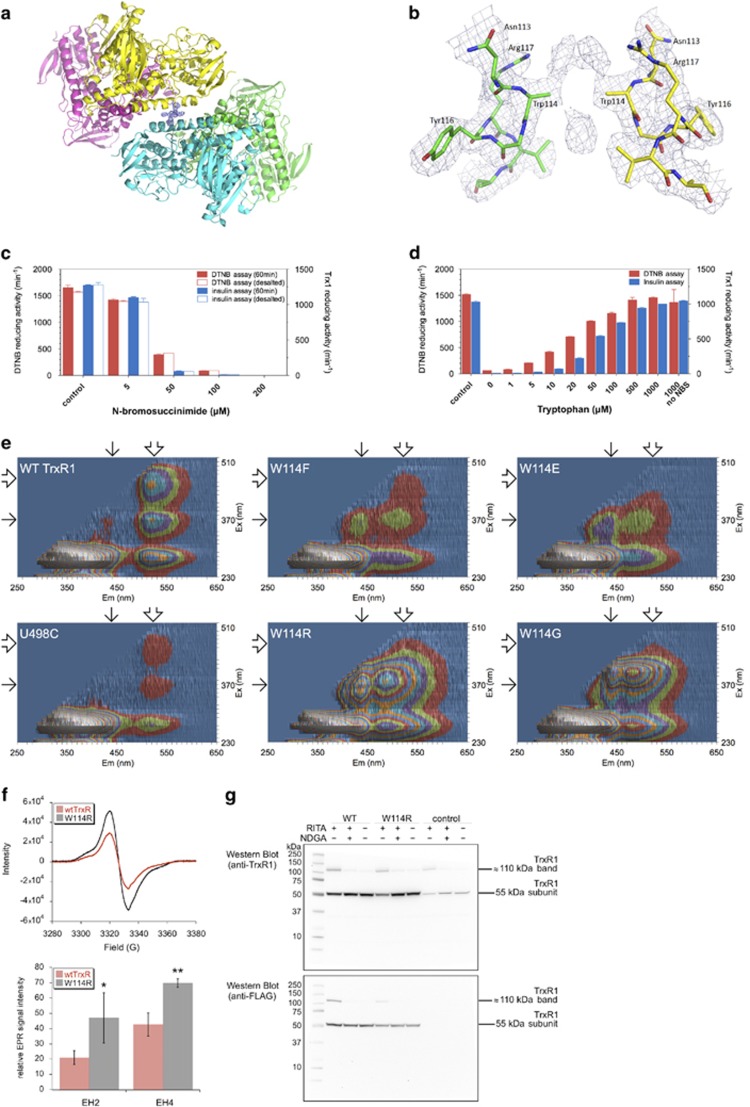
Surface-exposed Trp114 links TrxR1 dimers into tetramers, communicates with the FAD and is required for full enzyme activity. (**a**) Crystal structure of the TrxR1 tetramer, showing two dimers (yellow/purple and green/blue) linked together by the interaction of two modified Trp114 residues (middle, surface between the yellow and blue subunits of the two separate dimers; here with their modified side chains indicated by the composite omit electron density map). (**b**) Close-up of the final 2Fo-Fc map at 1σ of the region where two modified Trp114 side chains link the two TrxR1 dimers in the tetramer structure (one dimer here colored green and the other yellow). The electron density spanning the linkage could not be modeled with any known modifications of Trp residues and the Trp114 side chains are therefore only shown as electron density. Neighboring residues from each dimer are indicated with their three letter codes. (**c**) NBS irreversibly inhibits TrxR1. For this experiment, wild-type dimeric TrxR1 was incubated in the dark with NBS at the indicated concentrations for 60 min, desalted and subsequently enzyme activities were measured with either DTNB or Trx-dependent insulin reduction. (**d**) Free Trp in solution protects TrxR1 from inhibition by incubation for 120 min with 50 *μ*M NBS. (**e**) 3D fluorescence excitation/emission spectra reveal long-range communication between Trp114 and FAD. Typical enzyme-bound flavin fluorescence^53^ at Em_max_≈520 nm has three excitation peaks of Ex_max_≈280 nm, ≈370 nm and ≈470 nm as seen in wild-type TrxR1, whereas one of these fluorescence peaks, Ex_max_≈470 nm/Em_max_≈520 nm (block arrows), was virtually absent in the Trp114 variants, where instead a new peak at Ex_max_≈370 nm/Em_max_≈440 nm was seen (thin arrows). (**f**) Representative EPR analysis of wild-type TrxR and the W114R variant. For these spectra, TrxR (300 *μ*M dimer) was incubated with 900 *μ*M NADPH for 80 s. In the absence of NADPH, the oxidized enzymes did not show this signal, nor did NADPH without enzyme. The bar graph at right shows the relative EPR signal intensities of wild-type TrxR (wtTrxR) and the W114R variant incubated with an excess of NADPH to generate the reduced (EH4) state of the enzyme, or with a limiting amount of NADPH to generate a partially reduced (EH2) state. *P<0.05 or **P<0.01 for W114R *versus* wtTrxR. EPR instrument settings are described in the Materials and methods section. (**g**) Immunoblot detection of different TrxR1 species with antibodies against either TrxR1 (top) or the FLAG-tag (bottom) as resolved on reducing SDS-PAGE analyses with lysates from HCT116 cells transfected for expression of FLAG-tagged wild-type TrxR1 (WT), the W114R mutant or non-transfected controls, treated with either RITA, RITA and NDGA or using non-treated control cells (as indicated)

Tetrameric or other oligomeric species of TrxR1 are typically also found in trace amounts in preparations of the recombinant enzyme.^10^ Such tetramers were therefore, here, further purified to enable in-depth studies. The tetramers were found to be stable as tetramers in native PAGE under oxidized (non-reducing) conditions, while they resolved into monomeric subunits on SDS-PAGE and migrated as mainly dimeric TrxR1 on native PAGE under reducing conditions (Supplementary Figure S1 and S2). This suggested that the native non-covalently linked homodimeric form of TrxR1 easily oligomerizes into tetramers under oxidizing conditions and that some tetramers may also be covalently linked, presumably through oxidized forms of Trp114 (see Supplementary Scheme S1). To analyze the features of tetrameric TrxR1 in greater detail, we next purified high amounts of it in the absence of reductant and solved its crystal structure (PDB_4KPR, crystal data in Supplementary Table S2). The tetramer structure revealed that two modified Trp114 residues on the surfaces of two dimers were positioned toward each other, thereby linking two dimers into the tetrameric complex. Within this tetramer, the two dimers were composed of two non-covalently associated subunits in head-to-tail configuration, that is, the tetramer was a covalently linked complex of two otherwise nonmodified TrxR1 dimers (Figure 2a). The nature of the Trp114-mediated link was not consistent with known covalent linkages of oxidized tryptophan side chains, but the electron density showed clear interaction between the two dimers over two modified Trp114 residues (Figure 2b). Notably, this interaction and linkage between the dimers was the only major difference seen in the tetramer crystal structure, as compared with the previously characterized dimeric enzyme structure that was similarly packed in the crystal but lacked the link.^9^ Although the tetrameric form encompassed a linkage between two modified Trp114 residues, the two remaining Trp114 residues of the tetramer located at the surfaces not involved in the dimer–dimer interface, remained essentially intact as judged from the density. Subsequent mass spectrometric analyses also revealed that Trp114 residues in peptides obtained from TrxR1 tetramers were typically present in several oxidized states, including kynurenine and *N*-formylkynurenine derivatives, whereas such modifications were not found in peptides derived from dimeric enzyme not containing the covalent crosslink (Supplementary Table S3). These findings strengthened the notion that Trp114 oxidation promotes the oligomerization of TrxR1, and if excessively oxidized could form a covalent link between two TrxR1 dimers (Supplementary Scheme S1). As higher oligomers of TrxR1 were previously shown to have significantly lower specific activity than the dimeric enzyme,^10^ we next asked if the integrity of Trp114 is indeed important for TrxR1 catalysis. First, we analyzed whether the activity of pure dimeric TrxR1 would be affected by incubation with *N*-bromosuccinimide (NBS), a tryptophan-brominating compound. Treatment with NBS, in the absence of NADPH to retain the classical redox active centers of the enzyme in inert oxidized states,^3^ indeed effectively and irreversibly inactivated the enzyme (Figure 2c). This is, to our knowledge, the first demonstration of an efficient irreversible inhibitor of mammalian TrxR1 that acts in the absence of NADPH. Addition of free tryptophan protected the enzyme at stoichiometric amounts with relation to NBS, suggesting specificity in Trp targeting (Figure 2d). Because surface-exposed Trp residues are typically not conserved in evolution unless they fulfill important roles in either catalysis or protein–protein interfaces,^19^ we found it intriguing that the easily oxidized surface-exposed Trp114 of TrxR1 is highly conserved among TrxR isoenzymes of higher organisms (Supplementary Figure S3). This suggests that the high reactivity of Trp114 has a biological role. To further assess its importance, we next generated and analyzed TrxR1 variants in which Trp114 was changed to either aromatic (Phe), charged (Glu or Arg) or devoid of side chain (Gly) amino acids. All of these Trp114-substituted TrxR1 variants seemed to retain their overall secondary structure and stability, as displayed by similar CD spectra with predicted secondary structure elements (Supplementary Figure S4) and highly similar thermostability curves (Supplementary Figure S5). They also retained their Sec-containing active sites, as demonstrated using [^75^Se]-selenite autoradiography (Supplementary Figure S6) or NADPH-dependent targeting of Sec with 5-IAF (Supplementary Figure S7). Any major effects on catalysis inferred by these Trp114 mutations should thus mainly be related to the role of Trp114 itself.

We found that changing Trp114 into any other residue lowered the propensity of TrxR1 to form oligomers, with Phe114 retaining most of the capacity to oligomerize and Gly114 showing the least oligomerization (Supplementary Figure S8). Interestingly, in spite of its surface-exposed localization far away from the FAD, we found that the Trp114 residue communicated with the FAD because its removal changed the FAD-derived fluorescence of the enzyme, which was most easily illustrated with 3D fluorescence spectra (Figure 2e and Supplementary Figure S9). With Trp114 changed to either Glu, Arg or Gly, a fluorescence peak with Ex_max_≈470 nm/Em_max_≈520 nm seen in wild-type TrxR1, disappeared and instead a novel peak with Ex_max_≈370 nm/Em_max_≈440 nm appeared (Figure 2e). These alterations in the FAD fluorescence peaks demonstrated that Trp114 not only modulates the oligomeric state of TrxR1, or functions in facilitating binding of Trx1 as has been proposed,^20^ but also serves the function of an electron relay in communication with the FAD. Involvement in electron shuttling may indeed help explain the exaggerated reactivity of this residue and its increased propensity to oxidize. As the W114F variant, but not the other mutants, retained some of the native FAD-derived fluorescence spectrum (Figure 2e), we suggest that the electron relay function of Trp114 may occur through an electron transfer mechanism, similar to the shift effects of Trp-to-Phe substitutions in other electron-channeling enzymes.^21, 22, 23, 24, 25, 26^ To probe the possible involvement of radical species, we performed EPR analyses of wild-type TrxR1 and the W114R variant. When incubated with NADPH, both wild-type TrxR1 and the W114R generated EPR spectra (*g*=2.0044, line width of 14 G) when analyzed at X-band (9.35 GHz), although the signal was consistently larger (1.6- to 2.2-fold) with W114R (Figure 2f). The signal height did not vary with the time of incubation of TrxR1 with NADPH (45–180 s), suggesting a steady state level of radical. The second harmonics of these spectra were identical for wild-type TrxR1 and W114R (Supplementary Figure S9), suggesting that these two enzymes have the same radical species. The shoulders in the wings of the spectra (Figure 2f) are analogous to those seen with other flavin semiquinone radicals and represent ^14^N parallel hyperfine splitting.^27^ EPR analysis at 10 K (e.g., 2 G modulation at 0.62 mW power, or 0.5 G modulation at 0.2 mW power), or using Q band (35 GHz) did not reveal additional features. The *g* value and line width (Figure 2f) are consistent with a flavin anion semiquinone,^27^ and the second harmonics of the spectra also resemble those for flavin anion semiquinones.^27^ The spectral features are not, however, consistent with some other possible radicals; the signal line width is too narrow for tyrosyl radical, which is typically 20 G or larger,^28^ or for a neutral flavin radical at 19–21 G.^27^ The spectra can also not represent a Sec radical because the Sec-minus truncated variant exhibits the same spectrum (Supplementary Figure S9). The larger EPR signal with W114R (Figure 2f) is consistent with a higher percentage of FAD anion semiquinone, indicating that when Trp114 is absent the equilibrium of electron transfer shifts toward the FAD. These EPR data are thereby consistent with the shift in FAD fluorescence properties (Figure 2e), again suggesting that Trp114 affects electron transfer pathways within TrxR1 and communicates with the FAD (Figure 2f). We could not find any direct evidence for stable Trp-derived radical species, using the 9.3 GHz instrumentation at low temperatures (either 10 K or 100 K). Although we cannot exclude the possibility that there is a small Trp radical signal within the spectra (at low abundance relative to the flavin anion semiquinone), the fact that the W114R variant has a larger signal with identifcal features shows that it does not represent a Trp114 radical.

We next determined the kinetic parameters of the Trp114 mutant TrxR1 variants using well-characterized TrxR1 substrates. The substrate specificity of TrxR1 was significantly altered upon mutation of Trp114, as summarized in Table 1. The most notable results were seen in Trx1 reduction, with combinations of both lowered *k*_*cat*_ and increased *K*_*m*_ on mutations of Trp114. The activity was least affected with W114F, whereby all of the tested substrates became lowered to ~50% of wild-type TrxR1 in terms of efficiency (*k*_*cat*_*/K*_*m*_), except for juglone where *k*_cat_ increased ~1.5-fold whereas *K*_m_ was essentially unchanged (Table 1). The W114R and W114G mutations markedly lowered the activity with Trx1, yielding a *k*_*cat*_ of≈10% of wild-type TrxR1 whereas *K*_*m*_ increased ~4.5-fold, thereby resulting in efficiencies of only ≈2–3% of wild-type TrxR1. These findings clearly identify the surface-exposed Trp114 as a key residue for TrxR1 activity, and the W114E mutation essentially abolished Trx1 reduction capacity (Table 1). Importantly, all of the mutant variants still retained considerable capacity to reduce substrates other than Trx1, including DTNB, selenite, 9,10-phenanthrenequinone (PQ) and lipoamide (Table 1). As the reduction of juglone by TrxR1 was earlier shown to be supported in a Sec-independent manner and believed to occur mainly through the N-terminal -CVNVGC-/FAD motif of TrxR^3^ while the reduction of selenite^29^ or 9,10-phenanthrenequinone^30^ are completely dependent upon the C-terminal Sec-motif, these results demonstrated that the different Trp114 mutant proteins were all functional in terms of NADPH usage, FAD functionality as well as Sec-dependent catalysis, but gravely impaired for Trx1 reduction both in terms of *K*_*m*_ and *k*_*cat*_. Interestingly, using ICP-MS, we found that the W114E variant had the highest selenium content of all variants, with ≈90% Se per subunit. Thus, correlating turnover with Se content of the different enzyme variants, the catalytic importance of Trp114 became even more evident (Supplementary Table S4).

The exact catalytic mechanism of TrxR1, explaining its use of the C-terminal selenolthiol motif for reduction of the active site disulfide in Trx1, has long been debated.^8, 9, 31, 32, 33, 34^ The steps for the reductive half-reaction of TrxR1, as well as for the mechanism of attacking the Trx1 disulfide, have not yet been fully resolved. Recently the crystal structure of a complex between mutant human TrxR1 (C497S/U498C, i.e., with a C-terminal -GSCG motif instead of the wild-type -GCUG) and an active site mutant of human Trx1 (C73S/C35S) was described.^20^ The overall conformation of the mutant TrxR in that complex displayed no major differences to earlier published crystal structures of mammalian TrxR enzymes,^8, 9^ although the flexible C-terminal arm was surface-exposed and formed a mixed disulfide between Cys498 of mutant TrxR1 with Cys32 in mutant hTrx. The complex was suggested to illustrate a presumed transient selenenylsulfide-linked intermediate that would form during normal catalysis with native enzymes, whereby Trp114 was proposed to increase the affinity of TrxR1 for Trx1 as it resulted in a lower *K*_*m*_ value when Trp114 was present.^20^ Our results with a drastically reduced *k*_cat_ upon mutation of Trp114 in the genuine selenoprotein scaffold (Table 1) and the communication of Trp114 with the FAD (Figures 2e and f) suggest additional roles for Trp114 in catalysis than merely increasing affinity for Trx1. Furthermore, it cannot be ruled out that the use of two single-thiol mutant proteins ‘forced' into a complex as studied previously, might not necessarily be indicative of the native catalytic intermediate as proposed.^20^ Indeed, we found that several variants of disulfide-linked complexes between TrxR1 and Trx1 could be produced using different combinations of single-thiol active site mutants of the proteins (Supplementary Figure S10) and it cannot, therefore, be concluded which of those would resemble a true catalytic intermediate between the wild-type enzymes. We can conclude, however, that the integrity of the surface-exposed Trp114 residue is important for TrxR1 activity, mostly pronounced when coupled to Trx1 reduction.

We next probed whether the Trp-to-Arg substitution found here to affect both the oligomerization state of TrxR1 and the FAD fluorescence *in vitro*, would have effects on the RITA-induced formation of the covalently linked 110-kDa TrxR1 species in a cellular context. On the basis of the proposed importance of Trp114 for the crosslink, as suggested by the crystal structure (Figures 2a and b; Supplementary Scheme S1), this should be prevented if cells expressing Trp114 mutant enzyme would be treated with RITA. Indeed, with overexpression of N-terminally FLAG-tagged TrxR1 species, to enable targeted immunoblotting-based detection, we found that a Trp114 mutated variant clearly formed lower levels of crosslinked enzyme compared with the native protein (Figure 2g). Remaining traces of covalently linked dimers seen with the mutant may either be explained by crosslinks to subunits of the endogenously expressed native enzyme, or represent other forms of covalent complexes resistant to DTT and SDS, for example, containing diselenide linkages that were previously shown not to be resolved in reducing SDS-PAGE analyses.^35^ The results still showed that Trp114 is clearly important for RITA-triggered formation of covalently crosslinked subunits of TrxR1 in the cellular context, thus suggesting a mechanism similar to that characterized above with the purified and crystallized tetrameric form of TrxR1. It should be noted that not all oxidizing conditions in cells induce covalent crosslinking of TrxR1 subunits, as we could not see this phenomenon on treatment of cells with auranofin.^13^ However, it is promoted by iron–triapine (not shown), a pro-oxidant iron complex whose redox cycling is catalyzed by TrxR1.^36^ Interestingly, we have also previously discovered formation of covalent non-DTT reducible crosslinks of TrxR1 with either Trx1 or thioredoxin-related protein of 14 kDa (TRP14) on treatment of cells with platinum compounds, such as cisplatin.^37^ It is not yet clear whether a modified Trp114 residue was involved in the triggering of these other covalent complexes, or what cellular features that need to be fulfilled for different TrxR1 complexes to be formed. This should be the focus of future studies.

Tryptophan residues are often found in protein–protein interfaces along electron transfer pathways,^21, 22, 23, 24, 25, 26^ or in enzyme active sites where they can serve as mediators of electron transfer in peroxidases.^25, 38, 39^ On the basis of our present study, the role of Trp114 in modulating TrxR1 activity must be considered for the full understanding of cellular redox control. Here, we showed that Trp114 communicates with the FAD of TrxR1 and we probed how the integrity of the residue is required for efficient catalysis. As this surface-exposed Trp114 residue in oxidized form promotes oligomerization, thereby lowering the TrxR1 activity, this makes the residue perfectly poised to serve the role of an irreversible sensor for excessive oxidative stress. We thus propose that the evolutionary conservation of the reactive Trp114 residue in TrxR1 isoenzymes may potentially be explained by its increased capacity to react to oxidation, resulting in a lower activity of TrxR1. Such behavior suggests that if the thioredoxin system as a whole, in a cell or within a specific subcellular compartment as that during an oxidative burst,^6, 40^ cannot keep up with overly oxidizing conditions, then oxidation of the Trp114 residue in TrxR1 would promote a silencing of the thioredoxin system until new nonmodified TrxR1 species are made available, for example, by new synthesis of TrxR1. If such oxidation of Trp114 of TrxR1 would occur as a cellular global event and supersede new synthesis of nonmodified TrxR1 species, it could be a part of the triggering of cell death, as in the case with RITA treatment shown here. If this event instead would be localized to a signalosome, then compartmentalized TrxR1 silencing through Trp114 modification could assist oxidative-burst-related signaling.^40, 41^ The high reactivity and sensitivity to oxidation of Trp114 in TrxR1 can thereby have several roles in redox control and cellular signaling, which should clearly be the focus of future studies.

## Materials and Methods

For additional detailed protocols, please see the Supplementary Information.

### Expression and purification of recombinant TrxR1 variants

Mammalian rat TrxR1 and its mutants were expressed in the *Escherichia coli* BL21 (DE3) gor^−^ strains co-transformed with the pET-TRS_TER_-derived plasmids and the pSUABC plasmid according to the method of engineering a gene compatible with the bacterial selenoprotein synthesis machinery^42^ and using the '2.4/24/24' protocol, as described previously,^43^ except that a rich LB broth containing 10 g NaCl, 10 g peptone and 10 g yeast extract per liter was used. The TrxR1 U498C/W114 mutants and other non-Sec-containing variants were expressed in BL21 (DE3) gor^−^ strains, as described previously^44^ for the U498C mutant and the truncated forms. The soluble supernatant of TrxR1 samples was loaded onto the 2′5′-ADP Sepharose column (30 ml, GE Healthcare Life Sciences, Uppsala, Sweden) and protein purification was performed essentially as described before.^42^ The purified enzyme in TE buffer (pH 7.5) was concentrated by using 30-kDa cutoff Centrifugal filter device (Ultracel YM-30, Millipore, Billerica, MA, USA). Subsequent separation of rat TrxR1 oligomers was performed by size-exclusive chromatography – Superdex G200 column (GE Healthcare Life Sciences, Uppsala, Sweden) at 4 °C using an ÄKTA Explorer 100 workstation (GE Healthcare Life Sciences, Uppsala, Sweden), monitoring the absorbance simultaneously at both 280 nm (protein detection) and 463 nm (FAD detection). Flavoprotein concentration was determined by measuring the FAD absorbance at 463 nm (ɛ_FAD, 463 nm_=11 300 M^−1^ cm^−1^). The Bradford method was also used to estimate protein concentration and BSA was used as the standard (Bio-Rad, Hercules, CA, USA).

### Kinetic analyses of TrxR1 variants

Enzymatic activities of TrxR1 and its mutants were determined by using 5,5′-dithiobis-2-nitrobenzoic acid (DTNB) as a model substrate, and the formation of TNB^−^ was monitored as the increase in absorbance at 412 nm (ɛ_TNB, 412 nm_=13 600 M^−1^ cm^−1^). In all other assays (insulin-coupled thioredoxin reduction assay, juglone reduction assay, selenite reduction assay, 9,10-phenanthrenequinone reduction assay,^30^ lipoamide reduction assay), the oxidation of NADPH was monitored as the decrease in absorbance at 340 nm (ɛ_NADPH_, 340 nm=6200 M^−1^ cm^−1^). The standard reaction mixture (500 *μ*l) contained 200 (up to 300) *μ*M NADPH and 10–136 nM enzyme in 50 mM TE buffer (pH 7.5). The reaction was performed at 25 °C in a VersaMax spectrophotometer (Molecular Devices, Sunnyvale, CA, USA), using the same reaction mixture without enzyme as the reference. For each data point, the initial velocity was determined in triplicate over at least five different substrate concentrations. Control assays lacking the substrate were routinely included. Kinetic constants were calculated with Prism 5 software (GraphPad, San Diego, CA, USA) after direct plotting of the velocity *versus* substrate concentration followed by automatic Michaelis–Menten fit with nonlinear regression.

### Co-immunoprecipitation

HCT116 cells were cultured to a confluence of 80–90% in 196 cm^2^ dishes before treatment with 0.1% DMSO (three dishes) or 1 *μ*M RITA (six dishes) for 8 h. After washing with PBS, the cells were collected at 800 r.p.m. and resuspended in 700 *μ*l Extraction Buffer (50 mM Tris-HCl, 5 mM EDTA, 150 mM NaCl, 0.1% Tween-20, pH 7.5) per dish, followed by three cycles of freezing and rapid thawing. Cell debris was removed by centrifugation at 16 000 × *g* for 30 min and the protein concentrations of cell lysates were determined using the Bradford reagent (Bio-Rad, Hercules, CA, USA) and adjusted to 7 mg/ml. The resulting cleared lysates were used for co-immunoprecipitation experiments with anti-human TrxR1-19A1 monoclonal antibody. 675 *μ*l Dynabeads Protein G (Life Technologies, Carlsbad, CA, USA) were incubated with 135 *μ*l anti-TrxR1-19A1 antibodies and 450 *μ*l Extraction Buffer for 2 h at 4 °C. Then, 150 *μ*l of this mixture was subsequently added to each sample and incubated with agitation at 4 °C for 4 h. The samples were centrifuged at 27 000 × *g* for 10 s to collect the beads. A magnet was also used to aid in beads handling. The supernatant was removed and the beads were washed three times with 1 ml Extraction Buffer (see above) before elution by incubation with 60 *μ*l 0.1 M citrate buffer (pH 3.1) for 2 min. Then, 120 *μ*l neutralizing buffer (1 M Tris-HCl containing 3 mM EDTA, pH 7.5) was immediately added. The eluates of all three DMSO controls and two times of the three RITA-treated samples were combined and concentrated to 25 *μ*l using 30-kDa NMWL centrifugal filter units (Millipore). Two microliters of eluate was used for western blotting using anti-TrxR1 as the primary antibody, as described above. Horseradish peroxidase (HRP) anti-mouse IgG (Mouse TrueBlot ULTRA, 18-8817, eBioscience, San Diego, CA, USA) was used to minimize crossreactivity with the heavy and light chain IgG, present in all IP samples. In parallel, 20 *μ*l of each eluate was analyzed by 7% Tris-Acetate SDS-PAGE gels (Life Technologies, Grand Island, NY, USA) and stained afterwards by using EZBlue Gel Staining Reagent (Sigma, St. Louis, MO, USA).

### Mass spectrometry analyses

Bands of interest were cut out from the stained gel and treated for in-gel digestion as described.^45^ Briefly, the bands were de-stained using acetonitrile and ammonium bicarbonate, whereupon trypsin (porcine, modified, sequence grade, Promega, Madison, WI, USA) was introduced to the dried gel pieces. After overnight tryptic digestion, the peptides were analyzed by MALDI-TOF-MS on an Ultraflex I TOF/TOF from Bruker Daltonics (Bremen, Germany) using alfa-cyano 4-hydroxy cinnamic acid as matrix. The mass lists generated were used to scan for identity using the NCBI nr sequence database and the current version of the search engine ProFound (http://prowl.rockefeller.edu/prowl-cgi/ProFound). The spectrum was internally calibrated using autolytic tryptic peptides, and the error was set at ±0.07 Da. One missed cleavage site was allowed and methionine residues could be oxidized. Any potential tryptophan modifications were also analyzed. The significance of identity was judged from the search engine scoring system as well as other parameters such as similarity between empiric and calculated peptide masses.

### Crystallization, data collection and processing

Crystallization of purified tetrameric TrxR1 was performed by the hanging drop vapor diffusion method, using a precipitant solution containing 45% MPD (hexylene glycol, Sigma-Aldrich, St. Louis, MO, USA) and 100 mM Tris-HCl buffer (pH 7.6). Drops containing 2 *μ*l of protein (15 mg/ml, 7 U/mg) were mixed with 1 *μ*l of the precipitant solution at room temperature. Crystals were obtained after 5 days. X-ray data were collected under cryogenic conditions at beamline ID23-2 at ESRF, Grenoble, France at a wavelength of 0.8626 Å using a MAR225 detector. The diffraction data were processed with the program MOSFLM^46^ and scaled with SCALA from the CCP4 suite.^47^ Crystals of TrxR1 belong to the trigonal space group P3_1_2 with cell dimensions *a*=*b*=162.99 Å, *c*=236.44 Å. Data collection statistics are presented in Supplementary Table S2.

### Structure determination and refinement

Phasing by molecular replacement was performed using PHASER^48^ and the atomic coordinates of dimeric TrxR (PDB code 3EAN), stripped of all ligands and water. The molecular replacement solution contained four subunits per asymmetric unit. Models were built by alternating rounds of model-building in WinCoot^49^ and refinement against data in REFMAC5^50^ to 2.4 Å using local NCS restraints. One TLS segment per monomer was used during refinement. The modified linking Trp114 side chains could not be modeled with any certainty because no known modification of tryptophan fitted well to the electron density. Therefore, regular tryptophan in double conformation was inserted at the very end of refinement. Refinement results are presented in Supplementary Table S2. The crystallographic data have been deposited in the Protein Data Bank (entry 4KPR).

### 3D Fluorescence emission excitation spectrum analysis

Wild-type rat TrxR1 and its variants were dissolved in TE buffer (pH 7.5). The protein concentrations were adjusted to 6.8 *μ*M on the basis of the FAD absorbance at 463 nm. Two hundred microliters (total volume) of these TrxR1 samples were loaded into a flat-bottomed 96-wells black plate (Thermo Fisher Scientific, Waltham, MA, USA) for fluorescence measurements using the EnSpire Fluorescence Analyzer (PerkinElmer, Waltham, MA, USA). The emission spectrum ranging from 250 nm–650 nm was obtained by exiting the sample at a fixed excitation wavelength of 230 nm and subsequently the excitation wavelength was increased by 10-nm intervals up to 510 nm. The data obtained for all excitation wavelengths were used to plot the fluorescence emission spectra in 3D format, with ‘x axis' as the excitation wavelength (Ex), ‘y axis' as the emission wavelength (Em), and the ‘z axis' as fluorescence intensity. After subtracting the TE buffer background, TrxR1 samples have background fluorescence at Em_max_=380 nm when excited at Ex_max_=280 nm, and three typical flavin fluorescence signaling at Em_max_=520 nm when excited at Ex_max_=280 nm, Ex_max_=370 nm and Ex_max_=470 nm.

### Electron paramagnetic resonance

Preparations of purified recombinant wild-type TrxR and the W114R variant were concentrated using ultrafiltration to a final concentration of about 300 *μ*M (TrxR homodimer, which is equivalent to 600 *μ*M of the subunits). The enzyme concentration was verified by the absorbance of its FAD at 463 nm (extinction coefficient 11 300 M^–1^ cm^–1^). The oxidized enzyme did not have an EPR signal, whereas enzyme reduced with NADPH showed a distinct signal at *g*=2.0044 as described in the results. TrxR was reduced to the EH4 state (the maximal extent of reduction by NADPH) by incubation with 4.4 mol of NADPH per mol of TrxR dimer. TrxR was also examined after incubation with 3 mol of NADPH per mol of TrxR dimer, which is sufficient to generate a mix of partially reduced (EH2) and the EH4 state of the enzyme. Following incubation at room temperature (22 °C) in 4-mm quartz EPR tubes, the TrxR/NADPH mixtures were immersed in liquid nitrogen (77 K) and stored, typically for <1 week. EPR spectra were obtained at 120 K using a Bruker EMX spectrometer (Silberstreifen, Germany) with a Bruker temperature controlling system. Typical instrument settings were 9.348 GHz, 3300 G field set, 100 G sweep, 2 G modulation, 81.92 ms time constant, 81.92 ms conversion time, 83.88 ms sweep time, 6.32 × 10^5^ gain, 34 dB (79.7 *μ*W) microwave power, 9 scans. P_1/2_, the power at which the X-band signal height is one-half the expected unsaturated signal height, is 0.1 mW at 120 K. Some samples were also analyzed at liquid helium temperature (10 K) using a Bruker E500 ELEXSYS spectrometer with an Oxford Instruments ESR-9 helium flow cryostat (Oxfordshire, UK) and a Bruker DM0101 cavity. Some samples were also analyzed at Q band (35 GHz) using a Varian E-9 ESR Multifrequency Spectrometer equipped with a Q-band bridge. EPR spectra were confirmed in replicate experiments. The *g* values were determined by comparison to the 2,2-diphenyl-1-picrylhydrazyl radical which has a *g* value of 2.0036. Spectral features were also compared using the SUMSPEC program (a graphing and data analysis program available from the National Biomedical ESR Center at the Medical College of Wisconsin, Milwaukee) to generate the second harmonic.

### Preparation of plasmids encoding FLAG-tagged human TrxR1 variants

A vector encoding human TrxR1^51, 52^ was kindly provided by Dr. Anastasios E. Damdimopoulos (Karolinska Institutet, Sweden) that was utilized as template to amplify the complete DNA sequence of TrxR1 including the SECIS element at the 3′-untranslated region, using the forward primer hTrxR1-FLAG-f: 5′-GTGGTCTCGATGACGACGATAAGATGAACGGCCCTGAAGATCTTC and reverse primer hTrxR1-r: 5′-GTGGTCTCGGATCCCCATTTCTTGAATTCGCCAAATG. The resulting PCR product was purified, cleaved by *Eco31*I and ligated into an *Eco31*I-linearized pEGFP-N3 vector. The resulting plasmid was then used as template in a second PCR for introducion of an N-terminal FLAG-tag (amino acid sequence DYKDDDDK), which also inactivated the previous green fluorescent protein fusion partner of the original insert, thus generating the phTrxR1-wt-FLAG plasmid, using primer pair of EGFP-f: 5′-GTGGTCTCGGATCCATCGCCACCATGGTGAG-3′; and EGFP-FLAG-r: 5′-GTGGTCTCGTCATCCTTGTAATCCATGGTGGCGAATTCGAAGC-3′. The backbone of the phTrxR1-wt-FLAG plasmid was also used as template to generate the Trp114 to Arg114 mutated phTrxR1-W114R-FLAG plasmid, using the forward primer hTrxR1-W114R-f: 5′-GTGGTCTCTTGAATCGTGGCTACCGAGTAGCTCTGC-3′ and reverse primer hTrxR1-W114X-r: 5′-GTGGTCTCATTCAAAGAGCCAATGTGATTCTGTACAGCTTC-3′.

### Expression of FLAG-tagged hTrxR1 variants in HCT116 cells

HCT116 (human colon cancer, ATCC) cells were cultured at 37 °C in a humidified atmosphere with 5% CO_2_, using Iscove's Modified Dulbecco's Medium (IMDM, Sigma; I3390) supplemented with 10% fetal bovine serum (PAA Laboratories, GE Healthcare Life Sciences, Pasching, Austria), 2 mM glutamine, 100 units penicillin/ml and 100 *μ*g streptomycin/ml (Biochrom GmbH, Berlin, Germany). Cells were seeded one day before transfection in 56 cm^2^ dishes (Nunc 150350) at a density of 2 × 10^6^ cells/dish. The HCT116 cells were transfected with 20 *μ*g of the phTrxR1-wt-FLAG or phTrxR1-W114R-FLAG plasmids with 75 *μ*l OptiMEM and 40 *μ*l TurboFect diluted in 10 ml media per dish including 25 nM sodium selenite overnight. Untransfected cells used as controls were at the same time changed to fresh medium containing 25 nM sodium selenite. After subsequent culturing for 24 h, cells were either untreated or treated with 50 *μ*M NDGA for 1 h, followed by exposure to 1 *μ*M RITA for 8 h. Cells were collected by trypsination and lysed by incubation in Extraction Buffer whereupon lysates were cleared by centrifugation at 16 000 × *g* at 4 °C for 15 min. Protein concentration was determined by the Bradford method (Bio-Rad) using a standard curve with bovine serum albumin (BSA, Sigma, USA). Samples with 20 *μ*g total protein were analyzed using NuPAGE 4–12% Bis-Tris gels (Life Technologies, Carlsbad, CA, USA), with proteins electroblotted to a nitrocellulose membrane using a Dry-Blot system (program p3, 7 min; Life Technologies). The membrane was subsequently briefly incubated in Ponceau S solution (P7170, Sigma) and a picture was taken as loading control, confirming equal loading in all lanes. Subsequently, the membrane was washed with TBS-T for 2 min to remove the Ponceau S staining and blocked in 5% fat-free milk at room temperature for 1 h. After washing three times for 10 min with TBS-T, the membrane was incubated in 1% BSA containing either anti-TrxR1 (dilution 1 : 2000, v/v; 19A1, SantaCruz Biotechnology, Heidelberg, Germany) or anti-Flag M2 (dilution 1 : 2000, v/v; F1804, Sigma) primary antibodies overnight. The membrane was subsequently washed with TBS-T solution three times for 10 min and then transferred into 5% fat-free milk solution containing secondary goat anti-mouse IgG conjugated to horseradish peroxidase (dilution 1 : 2000, v/v) for 1 h at room temperature. The membrane was finally washed with TBS-T solution three times for 10 min and antigen–antibody binding was thereupon detected using a Western Lightning Chemiluminescence Reagent kit (PerkinElmer).

## Figures and Tables

**Table 1:** Steady-state kinetic parameters of TrxR1 variants

	**Recombinant rat TrxR1 variants**					
	**WT**	**W114F**	**W114R**	**W114E**	**W114G**	
*Trx1*^a^					
* k*_cat_ (min)	1130±21	596±8	106±2	< 3	102±1
* K*_m_ (*μ*M)	7.9±0.3	6.5±0.2	36.9±1.2	NA	37.2±0.9
* k*_cat_/*K*_m_ (min/*μ*M)	144	92	3	NA	3

*DTNB*^b^					
* k*_cat_ (min)	2548±66	1312±41	1192±23	327±20	1003±27
* K*_m_ (*μ*M)	212.8±15.9	207.1±18.9	101.5±5.9	309.7±47.6	263.7±18.9
* k*_cat_/*K*_m_ (min/*μ*M)	12	6	12	1	4

*Juglone*^c^					
* k*_cat_ (min)	797±32	1192±72	594±28	876±54	1361±105
* K*_m_ (*μ*M)	7.6±1.0	6.8±1.3	6.3±1.0	8.4±1.6	8.9±2.1
* k*_cat_/*K*_m_ (min/*μ*M)	105	176	95	104	153

*Selenite*^c^					
* k*_cat_ (min)	479±20	270.5±13	216 ±15	104±6	202±8
*K*_m_ (*μ*M)	2.6±0.4	1.9±0.4	1.1±0.4	1.4±0.3	1.6±0.3
* k*_cat_/*K*_m_ (min/*μ*M)	183	142	198	77	124

*9,10-PQ*^c^					
* k*_cat_ (min)	1718±30	959±21	844±37	309±9	785±21
* K*_m_ (*μ*M)	3.8±0.2	3.7±0.3	4.7±0.7	2.4±0.3	4.0±0.4
* k*_cat_/*K*_m_ (min/*μ*M)	458	260	179	130	199

*Lipoamide*^c^					
* k*_cat_ (min)	597±19	339±12	320±12	137±6	228±7
* K*_m_ (mM)	1.3±0.1	1.4±0.1	1.3±0.1	0.8±0.1	1.1±0.1
* k*_cat_/*K*_m_ (min/mM)	467	241	239	165	217

NA, not applicable

a Human wild-type Trx1 was used in the insulin-coupled Trx reduction assay

b *k*_cat_ per dimeric enzyme and *K*m were calculated following the formation of TNB^−^ at 412 nm at 25 °C

c *k*_cat_ per dimeric enzyme and *K*m were calculated following NADPH oxidation at 340 nm at 25 °C

## Abbreviations

Arg or R: arginine
CD: circular dichroism
Cys or C: cysteine
Cox: cyclooxygenase
DEDA: 7,7-dimethyl-(5Z,8Z)-eicosadienoic acid
DMSO: dimethyl sulfoxide
EH2: two-electron reduced states of the enzyme
EH4: four-electron reduced states of the enzyme
Gly or G: glycine
Glu or E: glutamic acid
DTNB: 5,5-dithiobis-(2-nitrobenzoic acid) or Ellman's reagent
DTT: 1,4-dithiothreitol
EGF: epidermal growth factor
Ex_max_: excitation max
Em_max_: emission max
EPR: electron paramagnatic resonance
5-IAF: iodoacetamidofluorescein
FAD: flavin adenine dinucleotide
HCT116: human colon cancer cells
HRP: horseradish peroxidase
ICP-MS: inductively coupled plasma mass spectrometry
IMDM: Iscoves's Modified Dulbecco's Medium
Indo: indomethacin
juglone: 5-hydroxy-1,4-naphthoquinone
MALDI-TOF-MS: matrix-assisted laser desorption/ionization time of flight mass spectrometry
MPD: hexylene glycol
NADPH: nicotinamide adenine dinucleotide phosphate
NBS: *N*-bromosuccinimide
NDGA: nordihydroguareric acid
MAFP: methyl arachidonyl fluorophosphonate
PQ: phenanthrenequinone
PBS: phosphate buffered saline
TBS-T: Tris-buffered saline with 0.05% Tween-20
PDB: Protein Data Bank
Phe or F: phenylalanine
PLA2: phospholipase A2
PM: pyridoxamine dihydrochloride
RITA: a small molecule (NSC652287) named from its function of reactivation of p53 and induction of tumor cell apoptosis
SA: salicylamine
SDS: sodium dodecyl sulfate
Se: selenium
Sec or U: selenocysteine
SECIS: selenocysteine insertion sequence
Ser or S: serine
Trp or W: tryptophan
Trx: thioredoxin
TrxR1: thioredoxin reductase 1
WT: wild type.

## References

[bib1] Arnér ES, Holmgren A. The thioredoxin system in cancer. Semin Cancer Biol 2006; 16: 420–426. 17092741 10.1016/j.semcancer.2006.10.009

[bib2] Mahmood DF, Abderrazak A, Khadija EH, Simmet T, Rouis M. The thioredoxin system as a therapeutic target in human health and disease. Antioxid Redox Signal 2013; 19: 1266–1303. 23244617 10.1089/ars.2012.4757

[bib3] Arnér ES. Focus on mammalian thioredoxin reductases—important selenoproteins with versatile functions. Biochim Biophys Acta 2009; 1790: 495–526. 19364476 10.1016/j.bbagen.2009.01.014

[bib4] Matsuzawa A, Ichijo H. Redox control of cell fate by MAP kinase: physiological roles of ASK1-MAP kinase pathway in stress signaling. Biochim Biophys Acta 2008; 1780: 1325–1336. 18206122 10.1016/j.bbagen.2007.12.011

[bib5] Lillig CH, Holmgren A. Thioredoxin and related molecules—from biology to health and disease. Antioxid Redox Signal 2007; 9: 25–47. 17115886 10.1089/ars.2007.9.25

[bib6] Winterbourn CC. Reconciling the chemistry and biology of reactive oxygen species. Nat Chem Biol 2008; 4: 278–286. 18421291 10.1038/nchembio.85

[bib7] Sun QA, Wu Y, Zappacosta F, Jeang KT, Lee BJ, Hatfield DL et al. Redox regulation of cell signaling by selenocysteine in mammalian thioredoxin reductases. J Biol Chem 1999; 274: 24522–24530. 10455115 10.1074/jbc.274.35.24522

[bib8] Zhong L, Arner ES, Holmgren A. Structure and mechanism of mammalian thioredoxin reductase: the active site is a redox-active selenolthiol/selenenylsulfide formed from the conserved cysteine-selenocysteine sequence. Proc Natl Acad Sci USA 2000; 97: 5854–5859. 10801974 10.1073/pnas.100114897 PMC18523

[bib9] Cheng Q, Sandalova T, Lindqvist Y, Arnér ESJ. Crystal structure and catalysis of the selenoprotein thioredoxin reductase 1. J Biol Chem 2009; 284: 3998–4008. 19054767 10.1074/jbc.M807068200

[bib10] Rengby O, Cheng Q, Vahter M, Jornvall H, Arner ES. Highly active dimeric and low-activity tetrameric forms of selenium-containing rat thioredoxin reductase 1. Free Radic Biol Med 2009; 46: 893–904. 19146949 10.1016/j.freeradbiomed.2008.12.017

[bib11] Gladyshev VN, Jeang K-T, Stadtman TC. Selenocysteine, identified as the penultimate C-terminal residue in human T-cell thioredoxin reductase, corresponds to TGA in the human placental gene. Proc Natl Acad Sci USA 1996; 93: 6146–6151. 8650234 10.1073/pnas.93.12.6146 PMC39204

[bib12] Issaeva N, Bozko P, Enge M, Protopopova M, Verhoef LG, Masucci M et al. Small molecule RITA binds to p53, blocks p53-HDM-2 interaction and activates p53 function in tumors. Nat Med 2004; 10: 1321–1328. 15558054 10.1038/nm1146

[bib13] Hedstrom E, Eriksson S, Zawacka-Pankau J, Arner ES, Selivanova G. p53-dependent inhibition of TrxR1 contributes to the tumor-specific induction of apoptosis by RITA. Cell Cycle 2009; 8: 3576–3583. 10.4161/cc.8.21.9977 19838062

[bib14] Weilbacher A, Gutekunst M, Oren M, Aulitzky WE, van der Kuip H. RITA can induce cell death in p53-defective cells independently of p53 function via activation of JNK/SAPK and p38. Cell Death Dis 2014; 5: e1318. 25010984 10.1038/cddis.2014.284 PMC4123078

[bib15] Shi Y, Nikulenkov F, Zawacka-Pankau J, Li H, Gabdoulline R, Xu J et al. ROS-dependent activation of JNK converts p53 into an efficient inhibitor of oncogenes leading to robust apoptosis. Cell Death Differ 2014; 21: 612–623. 24413150 10.1038/cdd.2013.186 PMC3950324

[bib16] Rundlöf A-K, Janard M, Miranda-Vizuete A, Arnér ESJ. Evidence for intriguingly complex transcription of human thioredoxin reductase 1. Free Radic Biol Med 2004; 36: 641–656. 14980707 10.1016/j.freeradbiomed.2003.12.004

[bib17] Hashimoto K, Panchenko AR. Mechanisms of protein oligomerization, the critical role of insertions and deletions in maintaining different oligomeric states. Proc Natl Acad Sci USA 2010; 107: 20352–20357. 21048085 10.1073/pnas.1012999107 PMC2996646

[bib18] Floriano-Sanchez E, Villanueva C, Medina-Campos ON, Rocha D, Sanchez-Gonzalez DJ, Cardenas-Rodriguez N et al. Nordihydroguaiaretic acid is a potent *in vitro* scavenger of peroxynitrite, singlet oxygen, hydroxyl radical, superoxide anion and hypochlorous acid and prevents *in vivo* ozone-induced tyrosine nitration in lungs. Free Radic Res 2006; 40: 523–533. 16551579 10.1080/10715760500419365

[bib19] Ma B, Elkayam T, Wolfson H, Nussinov R. Protein-protein interactions: structurally conserved residues distinguish between binding sites and exposed protein surfaces. Proc Natl Acad Sci USA 2003; 100: 5772–5777. 12730379 10.1073/pnas.1030237100 PMC156276

[bib20] Fritz-Wolf K, Kehr S, Stumpf M, Rahlfs S, Becker K. Crystal structure of the human thioredoxin reductase-thioredoxin complex. Nat Commun 2011; 2: 383. 21750537 10.1038/ncomms1382

[bib21] Tarboush NA, Jensen LM, Yukl ET, Geng J, Liu A, Wilmot CM et al. Mutagenesis of tryptophan199 suggests that hopping is required for MauG-dependent tryptophan tryptophylquinone biosynthesis. Proc Natl Acad Sci USA 2011; 108: 16956–16961. 21969534 10.1073/pnas.1109423108 PMC3193188

[bib22] Choi M, Shin S, Davidson VL. Characterization of electron tunneling and hole hopping reactions between different forms of MauG and methylamine dehydrogenase within a natural protein complex. Biochemistry 2012; 51: 6942–6949. 22897160 10.1021/bi300817d PMC3490227

[bib23] Byrdin M, Eker AP, Vos MH, Brettel K. Dissection of the triple tryptophan electron transfer chain in *Escherichia coli* DNA photolyase: Trp382 is the primary donor in photoactivation. Proc Natl Acad Sci USA 2003; 100: 8676–8681. 12835419 10.1073/pnas.1531645100 PMC166371

[bib24] Shih C, Museth AK, Abrahamsson M, Blanco-Rodriguez AM, Di Bilio AJ, Sudhamsu J et al. Tryptophan-accelerated electron flow through proteins. Science 2008; 320: 1760–1762. 18583608 10.1126/science.1158241

[bib25] Smith AT, Doyle WA, Dorlet P, Ivancich A. Spectroscopic evidence for an engineered, catalytically active Trp radical that creates the unique reactivity of lignin peroxidase. Proc Natl Acad Sci USA 2009; 106: 16084–16089. 19805263 10.1073/pnas.0904535106 PMC2752603

[bib26] Ivancich A, Dorlet P, Goodin DB, Un S. Multifrequency high-field EPR study of the tryptophanyl and tyrosyl radical intermediates in wild-type and the W191G mutant of cytochrome c peroxidase. J Am Chem Soc 2001; 123: 5050–5058. 11457334 10.1021/ja0036514

[bib27] Barquera B, Morgan JE, Lukoyanov D, Scholes CP, Gennis RB, Nilges MJ. X- and W-band EPR and Q-band ENDOR studies of the flavin radical in the Na+ -translocating NADH:quinone oxidoreductase from *Vibrio cholerae*. J Am Chem Soc 2003; 125: 265–275. 12515529 10.1021/ja0207201

[bib28] Rustandi RR, Jorns MS. Photoinduced spin-polarized radical pair formation in a DNA photolyase.substrate complex at low temperature. Biochemistry 1995; 34: 2284–2288. 7857939 10.1021/bi00007a024

[bib29] Kumar S, Bjornstedt M, Holmgren A. Selenite is a substrate for calf thymus thioredoxin reductase and thioredoxin and elicits a large non-stoichiometric oxidation of NADPH in the presence of oxygen. Eur J Biochem 1992; 207: 435–439. 1321713 10.1111/j.1432-1033.1992.tb17068.x

[bib30] Cenas N, Nivinskas H, Anusevicius Z, Sarlauskas J, Lederer F, Arner ES. Interactions of quinones with thioredoxin reductase: a challenge to the antioxidant role of the mammalian selenoprotein. J Biol Chem 2004; 279: 2583–2592. 14604985 10.1074/jbc.M310292200

[bib31] Zhong L, Holmgren A. Essential role of selenium in the catalytic activities of mammalian thioredoxin reductase revealed by characterization of recombinant enzymes with selenocysteine mutations. J Biol Chem 2000; 275: 18121–18128. 10849437 10.1074/jbc.M000690200

[bib32] Lothrop AP, Snider GW, Flemer Jr S, Ruggles EL, Davidson RS, Lamb AL et al. Compensating for the absence of selenocysteine in high-molecular weight thioredoxin reductases: the electrophilic activation hypothesis. Biochemistry 2014; 53: 664–674. 24490974 10.1021/bi4007258 PMC3931472

[bib33] Snider GW, Dustin CM, Ruggles EL, Hondal RJ. A mechanistic investigation of the C-terminal redox motif of thioredoxin reductase from *Plasmodium falciparum*. Biochemistry 2014; 53: 601–609. 24400600 10.1021/bi400931k PMC3957191

[bib34] Lothrop AP, Snider GW, Ruggles EL, Hondal RJ. Why is mammalian thioredoxin reductase 1 so dependent upon the use of selenium? Biochemistry 2014; 53: 554–565. 24393022 10.1021/bi400651x PMC3957196

[bib35] Shchedrina VA, Novoselov SV, Malinouski MY, Gladyshev VN. Identification and characterization of a selenoprotein family containing a diselenide bond in a redox motif. Proc Natl Acad Sci USA 2007; 104: 13919–13924. 17715293 10.1073/pnas.0703448104 PMC1955791

[bib36] Myers JM, Cheng Q, Antholine WE, Kalyanaraman B, Filipovska A, Arner ES et al. Redox activation of Fe(III)-thiosemicarbazones and Fe(III)-bleomycin by thioredoxin reductase: specificity of enzymatic redox centers and analysis of reactive species formation by ESR spin trapping. Free Radic Biol Med 2013; 60: 183–194. 23485585 10.1016/j.freeradbiomed.2013.02.016 PMC3654041

[bib37] Prast-Nielsen S, Cebula M, Pader I, Arner ES. Noble metal targeting of thioredoxin reductase—covalent complexes with thioredoxin and thioredoxin-related protein of 14 kDa triggered by cisplatin. Free Radic Biol Med 2010; 49: 1765–1778. 20851179 10.1016/j.freeradbiomed.2010.09.008

[bib38] Stubbe J, Nocera DG, Yee CS, Chang MC. Radical initiation in the class I ribonucleotide reductase: long-range proton-coupled electron transfer? Chem Rev 2003; 103: 2167–2201. 12797828 10.1021/cr020421u

[bib39] Morimoto A, Tanaka M, Takahashi S, Ishimori K, Hori H, Morishima I. Detection of a tryptophan radical as an intermediate species in the reaction of horseradish peroxidase mutant (Phe-221 —> Trp) and hydrogen peroxide. J Biol Chem 1998; 273: 14753–14760. 9614074 10.1074/jbc.273.24.14753

[bib40] Finkel T. Signal transduction by reactive oxygen species. J Cell Biol 2011; 194: 7–15. 21746850 10.1083/jcb.201102095 PMC3135394

[bib41] Lee S, Kim SM, Lee RT. Thioredoxin and thioredoxin target proteins: from molecular mechanisms to functional significance. Antioxid Redox Signal 2012; 18: 1165–1207. 22607099 10.1089/ars.2011.4322 PMC3579385

[bib42] Böck A, Forchhammer K, Heider J, Leinfelder W, Sawers G, Veprek B et al. Selenocysteine: the 21st amino acid. Mol Microbiol 1991; 5: 515–520. 1828528 10.1111/j.1365-2958.1991.tb00722.x

[bib43] Rengby O, Johansson L, Carlson LA, Serini E, Vlamis-Gardikas A, Karsnas P et al. Assessment of production conditions for efficient use of *Escherichia coli* in high-yield heterologous recombinant selenoprotein synthesis. Appl Environ Microbiol 2004; 70: 5159–5167. 15345395 10.1128/AEM.70.9.5159-5167.2004 PMC520894

[bib44] Anestal K, Prast-Nielsen S, Cenas N, Arner ES. Cell death by SecTRAPs: thioredoxin reductase as a prooxidant killer of cells. PLoS One 2008; 3: e1846. 18382651 10.1371/journal.pone.0001846 PMC2268967

[bib45] Hellman U. Peptide mapping using MALDI-TOFMS. In: Silberring J, EkmanR (eds). Mass Spectrometry and Hyphenated Techniques in Neuropeptide Research Wiley. Wiley: Hoboken, NJ, USA, 2002; pp 259–275.

[bib46] Leslie AG. The integration of macromolecular diffraction data. Acta Crystallogr D Biol Crystallogr 2006; 62: 48–57. 16369093 10.1107/S0907444905039107

[bib47] Winn MD, Ballard CC, Cowtan KD, Dodson EJ, Emsley P, Evans PR et al. Overview of the CCP4 suite and current developments. Acta Crystallogr D Biol Crystallogr 2011; 67: 235–242. 21460441 10.1107/S0907444910045749 PMC3069738

[bib48] McCoy AJ, Grosse-Kunstleve RW, Adams PD, Winn MD, Storoni LC, Read RJ. Phaser crystallographic software. J Appl Crystallogr 2007; 40: 658–674. 19461840 10.1107/S0021889807021206 PMC2483472

[bib49] Emsley P, Lohkamp B, Scott WG, Cowtan K. Features and development of Coot. Acta Crystallogr D Biol Crystallogr 2010; 66: 486–501. 20383002 10.1107/S0907444910007493 PMC2852313

[bib50] Murshudov GN, Vagin AA, Dodson EJ. Refinement of macromolecular structures by the maximum-likelihood method. Acta Crystallogr D Biol Crystallogr 1997; 53: 240–255. 15299926 10.1107/S0907444996012255

[bib51] Xia L, Nordman T, Olsson JM, Damdimopoulos A, Bjorkhem-Bergman L, Nalvarte I et al. The mammalian cytosolic selenoenzyme thioredoxin reductase reduces ubiquinone. A novel mechanism for defense against oxidative stress. J Biol Chem 2003; 278: 2141–2146. 12435734 10.1074/jbc.M210456200

[bib52] Nalvarte I, Damdimopoulos AE, Nystom C, Nordman T, Miranda-Vizuete A, Olsson JM et al. Overexpression of enzymatically active human cytosolic and mitochondrial thioredoxin reductase in HEK-293 cells. Effect on cell growth and differentiation. J Biol Chem 2004; 279: 54510–54517. 15471857 10.1074/jbc.M408494200

[bib53] Munro AW, Noble MA. Fluorescence analysis of flavoproteins. Methods Mol Biol 1999; 131: 25–48. 10494540 10.1385/1-59259-266-X:25

