# Supplementary material for: The conserved Trp114 residue of thioredoxin reductase 1 has a redox sensor-like function triggering oligomerization and crosslinking upon oxidative stress related to cell death
Source: Cell Death Dis. 2015 Jan 22;6(1):e1616–. doi: 10.1038/cddis.2014.574 (PMC4669772; doi:10.1038/cddis.2014.574)
Supplement: Supplementary Information [file cddis2014574x1.pdf]

Supplementary Material

The conserved Trp114 residue of thioredoxin reductase 1 has a redox sensor-like function triggering oligomerisation and crosslinking upon oxidative stress related to cell death

Jianqiang Xu[1§], Sofi E. Eriksson[1], Marcus Cebula[1], Tatyana Sandalova[2], Elisabeth Hedström[3], Irina Pader[1], Qing Cheng[1], Charles R. Myers[4], William E. Antholine[5], Péter Nagy[6], Ulf Hellman[7], Galina Selivanova[3], Ylva Lindqvist[2], Elias S. J. Arnér[1,]*

[1] Division of Biochemistry, Department of Medical Biochemistry and Biophysics, Karolinska Institutet, SE-171 77, Stockholm, Sweden.

[2] Division of Molecular Structural Biology, Department of Medical Biochemistry and Biophysics, Karolinska Institutet, SE-171 77, Stockholm, Sweden.

[3] Department of Microbiology, Tumor and Cell Biology, Karolinska Institutet, SE-171 77, Stockholm, Sweden.

[4] Department of Pharmacology and Toxicology, Medical College of Wisconsin, 8701 Watertown Plank Road, Milwaukee, WI 53226, USA.

[5] Department of Biophysics, Medical College of Wisconsin, 8701 Watertown Plank Road, Milwaukee, WI 53226, USA.

[6] Department of Molecular Immunology and Toxicology, The National Institute of Oncology, 1122 Budapest, Rath Gyorgy ut 7-9, Hungary.

[7] Ludwig Institutet for Cancer Research Ltd., Uppsala University BMC, SE-75 124, Uppsala, Sweden.

[§] Present affiliation: School of Life Science and Medicine, Dalian University of Technology, Panjin 124221, China

* To whom correspondence should be addressed. Tel.: 46-8-5248-69-83; Fax: 46-8-31-15-51; E-mail: Elias.Arner@ki.se.

This file includes:

Supplementary Methods and Detailed Protocols

Supplementary Scheme S1.

Supplementary Figures S1 to S10.

Supplementary Tables S1 to S4.

Supplementary Methods and Detailed Protocols

Cell culture, proliferation and viability assay

Human colon carcinoma HCT116 cells were received from B. Vogelstein, The Johns Hopkins University, USA by way of Stig Linder and Galina Selivanova (Karolinska Institutet, Sweden) and were maintained in Iscoves's Modified Dulbecco's Medium (IMDM, Sigma-Aldrich Chemicals) supplemented with 10 % fetal bovine serum, 2 mM L-glutamine, 100 U/ml penicillin, and 100 µg/ml streptomycin (PAA Laboratories, Austria). Cells were grown at 37 °C in an atmosphere of 5% CO_2 and humidified air. For experiments, cells were seeded in suitable plates, typically reaching a 60-70% confluence, approx. 15-18 h prior to the addition of drugs. During drug treatment, the final concentration in culture medium did not exceed 0.1% DMSO or 0.2% ethanol (v/v). The cellular reduction potential of tetrazolium salts, 3-(4,5-dimethylthiazolyl-2)-2,5-diphenyltetrazolium bromide (MTT) to formazans, was used to measure cell viability. Cells were plated in 96-well plates at a density of 1×10^4 cells/well (final volume of 80 µl) and incubated for 24 h with RITA, +/- NDGA preincubation. Controls received corresponding amounts of DMSO. After the treatment, cells were washed once with warm PBS and subsequently incubated at 37 °C with 200 µl fresh medium containing MTT. 2 mg/ml MTT dissolved in PBS was filter-sterilized before use at a working concentration of 0.5 mg/ml. After incubation for 4 h, the MTT-containing medium was carefully removed. 160 µl DMSO and 40 µl glycine buffer containing 100 mM glycine and 100 mM NaCl (pH 10.5), were added to each well to dissolve the newly formed formazan crystals. Then the plates were read at 550 nm at 25 °C in a VersaMax spectrophotometer (Molecular Devices, USA), using the same reaction mixture without cells as reference.

For the experiments with effects of several different antioxidants upon formation of the ≈ 110 kDa band of TrxR1 (**Figs. 1b,c**, main text), cells were preincubated with compounds before addition of RITA (for 60 min or 15 h, as indicated in **Fig. 1b**, or 45 min in **Fig. 1c**), with either NDGA, BW‑A‑4c, MAFP, DEDA, Indo, PM or SA, at concentrations as indicated in the figure legends. Thereafter the compounds were also included during an additional 8h incubation with RITA (except for one sample that was only preincubated with NDGA for 1 h and subsequently treated with RITA, as indicated in **Fig. 1b**). Medium was changed before addition of RITA and compounds. For the siRNA treatment, approx. 5×10^4 cells were seeded one day (15 h) before siRNA transfection, in which case RITA was added approx. 60 h after seeding.

Preparation of cell extracts and immunoblotting

Cells were harvested by trypsination and were then lysed by freezing and thawing in Extraction Buffer, containing 50 mM Tris-HCl (pH 7.5), 5 mM EDTA, 0.15 M NaCl, 1% Trition X-100, and

complete EDTA-free protease inhibitor cocktail (Roche, USA). Lysates were cleared by centrifugation at 16,000 × g at 4 °C for 10 min. Protein concentration was determined by the Bradford method (Bio-Rad, USA) using bovine serum albumin (BSA, Sigma, USA) as a standard. Samples with 20 μg of total protein (cell extracts purified by the ÄKTA, 15 μl fraction), were analyzed using NuPAGE 4-12% Bis-Tris gels (Life Technologies, USA). Three antibodies, mouse monoclonal TrxR1 (19A1), mouse monoclonal p53 (DO-1) and rabbit polyclonal glyceraldehyde-3-phosphate, GAPDH (FL-335) were used for western blot analysis (SantaCruz Biothechnology, Germany).

Transient knockdown of TrxR1 and ^{75}Se labeling of cellular proteins

HCT116 cells (5 × 10^4 cells/well) were seeded in 6-well plates. For small interfering RNA (siRNA) transfection, cells were incubated with 10 nM siRNA duplexes for 24 h, TrxR1 siRNA sequences (Siseq1 and Siseq2) and the scrambled control (mock) used. Transient transfection procedures have been previously described in (Eriksson et al, 2009). The indicated drugs were added 48 h post siRNA transfection. However, for the ^{75}Se-incorporation experiment, approx. 1 × 10^5 cells/well were plated in the presence of 1.5 μCi [^{75}Se]-selenite. After 24 h siRNA transfection, the medium was removed and fresh medium without ^{75}Se was added. Cells were subsequently incubated with the indicated drugs.

Endpoint insulin assay of cellular samples

The end-point Trx-dependent insulin assay (Arnér & Holmgren, 2001) was modified and applied to 96-well plates to measure the thioredoxin reductase activity of cell lysates. Briefly, 10 μl of fractions was incubated with 20 μM of wild type human Trx1, 297 μM insulin, and 1.3 mM NADPH, in presence of 85 mM HEPES buffer containing 13 mM EDTA (pH 7.5) at 37 °C for 90 min, in a total volume of 50 μl. The reaction was stopped by addition of 200 μl 7.2 M guanidine-HCl in 200 mM Tris-HCl, pH 8.0, containing 1 mM DTNB. The Trx-dependent newly formed thiols in the reduced insulin products were then determined at 25 °C by measuring the absorbance at 412 nm (extinction coefficient of 13,600 M^{-1} cm^{-1}) using a VersaMax spectrophotometer (Molecular Devices, USA) with a background absorbance reference for each samples containing all components except Trx, incubated and treated in the same manner.

Site-directed mutagenesis of recombinant rat TrxR1

Wild-type TrxR1 was generated as previously described using a recombinant system that provides for incorporation of the active site Sec (Böck et al, 1991). Several previously characterized site-directed mutants of TrxR1 that have been studied earlier, such as C59S (Cenas et al, 2004), C64S (Cenas et al, 2004), C59S/C64S (Anestal et al, 2008), U498C (Zhong et al, 2000), Y116I and

Y116T(Cheng et al, 2009), C189S and C189A (Xu & Arner, 2012), were also used in this study for comparison purposes. The UGA-truncated variant ending with -$Sec^{498}Gly^{499}$–COOH was produced as previously described (Anestal et al, 2008; Anestål & Arnér, 2003). The plasmid pET-TRS_{TER} constructed earlier (Böck et al, 1991) was prepared as template for all PCRs reactions to make W114 mutants, including W114F, W114R, W114E and W114G. The plasmid pET-TRS_{TER} (TGA-to-TGC mutation) (Zhong et al, 2000) was prepared as template for W114 double mutation. The forward primers were as follows: W114F-f, 5'-gcggtctcctgaacTTTggctaccgagtagctctc-3'; W114R-f, 5'-gcggtctcctgaacCGTggctaccgagtagctctc-3'; W114E-f, 5'-gcggtctcctgaacGAAggctaccgagtagctctc-3'; and W114G-f, 5'-gcggtctcctgaacGGCggctaccgagtagctctc-3'. The reverse primer was W114-r, 5'-gcggtctcgttcagcgagccgatatg-3'. In addition, ten new C-terminal mutants of TrxR1 were also made for further complex analysis, including -GUCG, -GSUG, -GUSG, -GSCG, -GCSG, -GSSG, -GU, -GS, -GC, and -G. The forward primers were as follows,

GUCG-f, 5'-gcggtctccagtccggaTGATGCggctaatcggttgcagg-3';
GSUG-f, 5'-gcggtctccagtccggaTCATGAggctaataatcggttgc-3';
GUSG-f, 5'-gcggtctccagtccggaTGATCAggctaatcggttgcagg-3';
GSCG-f, 5'-gcggtctccagtccggaTCATGCggctaataatcggttgc-3';
GCSG-f, 5'-gcggtctccagtccggaTGCTCAggctaataatcggttgc-3';
GSSG-f, 5'-gcggtctccagtccggaTCATCAggctaataatcggttgc-3';
GU-f, 5'-gcggtctccagtccggaTGATAAggctaataatcggttgc-3';
GS-f, 5'-gcggtctccagtccggaTCATAAggctaagcc-3';
GC-f, 5'-gcggtctccagtccggatgcTAAggctaagccccagaatc-3'; and
G-f, 5'-gcggtctccagtccggaTAAtaaggctaagcc-3'.

The reverse primer was C-term-r, 5'-gcggtctcggactggaggatgtctcc-3'. Phusion® High-Fidelity PCR Master Mix (Thermo Fisher Scientific, Waltham, USA) was utilized for the inverse PCRs and all molecular cloning was performed as described (Xu & Arner, 2012). In brief, together with the addition of 20 pmol of each primer and 10 ng of template plasmid in a total volume of 50 μl, PCR reactions were initiated at 98 °C for 2 min, followed by 30 cycles of amplification (98 °C, 10 s; 65 °C, 45 s; 72 °C, 3 min) and extended at 72 °C for additional 10 min. The resulting PCR products were analyzed by 1% agarose electrophoresis and the targeted PCR band was cut and then purified using the Gel Extraction Kit (Qiagen, Germany), digested with *Eco*31 I and *Dpn* I (Thermo Fisher Scientific, Waltham, USA) at 37 °C for 30 min, re-purified with the PCR purification kit (Qiagen, Germany), and ligated using T4 DNA ligase (Thermo Fisher Scientific, Waltham, USA) at 22 °C for 2 h. Finally, the DNA constructs were then transformed into *E. coli* BL21 (DE3) gor^- competent cells (Tet^+), and then DNA sequencing (GATC Biotech, Konstanz, Germany) verified the mutations.

Electrophoresis

TrxR1 samples were analyzed by SDS PAGE and native PAGE using either NuPAGE™ 4-12% Bis-Tris SDS gel or NativePAGE™ 4-16% Bis-Tris gel (Life Technologies, USA). The protein bands were stained using PhastGel™ Brilliant Blue R250 (GE Healthcare Life Sciences, Uppsala, Sweden), destained with 10% acetic acid and 30% methanol and then documented using a Bio-Rad ChemiDoc XRS scanner (Bio-Rad, USA). Band intensity analysis was performed using the 1-D Image Analysis software (Bio-Rad, USA).

Western blotting

Protein bands in the PAGE gel were electroblotted to a nitrocellulose membrane (Millipore, USA) at 30 V at 4 °C for 3 h. The membrane was blocked in 5% fat-free milk at 4 °C for 1 h and incubated in 1% BSA containing the anti-TrxR1 19A1 monoclonal antibody (dilution 1:1000, v/v) for 2 h. It was then washed with PBS-T solution (PBS, 0.1% Tween-20, pH 7.5) for 3 times of 5 min each, and then transferred into 1% BSA containing secondary goat anti-mouse IgG conjugated to horseradish peroxidase (dilution 1:500, v/v) for 1 h. The membrane was washed with PBS-T solution 3 times (5 min each). Finally, antigen-antibody binding was detected using the Western Lightning™ Chemiluminescence Reagent kit (Perkin Elmer, USA).

^{75}Se Autoradiography

Radiolabeling using 1 µCi [^{75}Se]-selenite was utilized for visualization of Sec incorporation into recombinantly expressed protein, using previously described protocols (Böck et al, 1991). 0.5 mM IPTG-induced protein samples were prepared and used for analyses with reducing SDS-PAGE gel and autoradiography with documentation using a GE Typhoon™ FLA 7000 Biomolecular Imagers and ImageQuant™ TL software version 7.0 (GE Healthcare Life Sciences, Uppsala, Sweden).

TrxR activity assays using 96-well microtiter plate

Enzymatic activities of purified TrxR1 variants were determined using four different assays in 96-well microtiter plate format. ***a)*** **Insulin-coupled Trx reduction assay** (Arnér & Holmgren, 2001). The standard reaction mixture (200 µl) contained 20 µM hTrx1, 160 µM insulin, 170 nM TrxR variant enzyme and 300 µM NADPH in 50 mM TE buffer (pH 7.5). Activity assay was performed following the NADPH consumption as decrease of A340 nm utilizing an extinction coefficient of 6,200 $M^{-1}cm^{-1}$. ***b)*** **DTNB reduction assay** (Arnér & Holmgren, 2001). The standard reaction mixture (200 µl) contained 2.5 mM DTNB, 17 nM TrxR variant enzyme, and 300 µM NADPH in 50 mM TE buffer, pH7.5. DTNB reduction was evaluated by the formation of TNB^- following the absorbance at 412 nm for 10 min, with the extinction coefficient of 13,600 $M^{-1}cm^{-1}$. ***c)*** **Juglone reduction assay** (Anestal et al, 2008; Anestål & Arnér, 2003). The standard reaction mixture (200 µl) contained 30 µM juglone, 17 nM TrxR variant enzyme and 200 µM NADPH in 50 mM TE

buffer (pH 7.5). The activity assay was performed following NADPH consumption as the decrease at A340 nm (extinction coefficient of 6,200 $M^{-1}cm^{-1}$). **d) 9,10-Phenanthrenequinone reduction assay** (Cenas et al, 2004). The standard reaction mixture (200 µl) in this assay contained 30 µM 9.10-phenanthrenequinone, 17 nM enzyme, and 200 µM NADPH in 50 mM TE buffer (pH 7.5) and activity was measured by following NADPH consumption at 340 nm. All the activity assays were performed with 10-sec time interval reads at 25 °C using a VersaMax microplate reader (Molecular Devices, USA), with the reaction mixtures without enzyme serving as reference. Activity measurements were performed in at least duplicate and analyzed with the Prism 5 software (GraphPad, USA).

Circular Dichroism (CD) Spectrum Analysis

Circular dichroism spectrum analysis was carried out with a 1 cm quartz cuvette at 20 °C using a Jasco™ J-810 Spectropolarimeter with Jasco™ PTC-423S single position Peltier thermostatted cell holder (Jasco, Japan). Each sample (800 µl total volume) contained 100 nM TrxR1 variants in 1 mM Tris-HCl buffer containing 40 nM EDTA (pH 7.5). The CD spectra scanning were scanned from 195 nm to 260 nm with a step size of 1 nm. The buffer spectrum was subtracted from the sample spectra. Secondary structure calculations were done with DICHROWEB (http://dichroweb.cryst.bbk.ac.uk/) for circular dichroism spectral analysis based on the K2d method.

Thermostability Analysis

Thermostability of TrxR1 variants was measured in parallel by applying a light-scattering-based methodology (Vedadi et al, 2006) with a multi-well format StarGazer-384™ instrument (Harbinger Biotechnology and Engineering Corp., Toronto, Canada). TrxR1 samples in flat-bottomed 384-well black plates were subjected to gradually increased temperature from 25 °C to 80 °C at 1 °C per min. 30 µl enzyme solution was placed inside of each well and then mixed with 20 µl TE buffer (pH 7.5). Mineral oil (40 µl) (M1180, Sigma, USA) was added to the wells to protect sample evaporation. Images of scattered light were taken every 30 s to monitor protein aggregation.

Selenium Determination

Selenium contents of TrxR1 variants were determined by elemental analysis (ALS Scandinavia AB, Luleå, Sweden), using two separate samples per enzyme. The results were used for normalization of catalytic turnover numbers with regards to selenium content as described in Table E4.

References for the supplementary methods section:

Anestal K, Prast-Nielsen S, Cenas N, Arner ES (2008) Cell death by SecTRAPs: thioredoxin reductase as a prooxidant killer of cells. *PLoS ONE* **3:** e1846

Anestål K, Arnér ES (2003) Rapid induction of cell death by selenium-compromised thioredoxin reductase 1 but not by the fully active enzyme containing selenocysteine. *J Biol Chem* **278:** 15966-15972

Arnér ES, Holmgren A (2001) Measurement of thioredoxin and thioredoxin reductase. *Curr Protoc Toxicol* **Chapter 7:** Unit 7 4

Böck A, Forchhammer K, Heider J, Leinfelder W, Sawers G, Veprek B, Zinoni F (1991) Selenocysteine: the 21st amino acid. *Molecular microbiology* **5:** 515-520

Cenas N, Nivinskas H, Anusevicius Z, Sarlauskas J, Lederer F, Arner ES (2004) Interactions of quinones with thioredoxin reductase: a challenge to the antioxidant role of the mammalian selenoprotein. *J Biol Chem* **279:** 2583-2592

Cheng Q, Sandalova T, Lindqvist Y, Arnér ESJ (2009) Crystal structure and catalysis of the selenoprotein thioredoxin reductase 1. *J Biol Chem* **284:** 3998-4008

Emsley P, Lohkamp B, Scott WG, Cowtan K (2010) Features and development of Coot. *Acta crystallographica Section D, Biological crystallography* **66:** 486-501

Eriksson SE, Prast-Nielsen S, Flaberg E, Szekely L, Arner ES (2009) High levels of thioredoxin reductase 1 modulate drug-specific cytotoxic efficacy. *Free Radic Biol Med* **47:** 1661-1671

Hellman U (2002) Peptide mapping using MALDI-TOFMS. In *Mass spectrometry and hyphenated techniques in neuropeptide research.* , Silberring J, Ekman R (eds), pp pp 259-275 Wiley: Hoboken, NJ, USA

Leslie AG (2006) The integration of macromolecular diffraction data. *Acta crystallographica Section D, Biological crystallography* **62:** 48-57

McCoy AJ, Grosse-Kunstleve RW, Adams PD, Winn MD, Storoni LC, Read RJ (2007) Phaser crystallographic software. *Journal of applied crystallography* **40:** 658-674

Murshudov GN, Vagin AA, Dodson EJ (1997) Refinement of macromolecular structures by the maximum-likelihood method. *Acta Crystallogr D Biol Crystallogr* **53:** 240-255

Nalvarte I, Damdimopoulos AE, Nystom C, Nordman T, Miranda-Vizuete A, Olsson JM, Eriksson L, Bjornstedt M, Arner ES, Spyrou G (2004) Overexpression of enzymatically active human cytosolic and mitochondrial thioredoxin reductase in HEK-293 cells. Effect on cell growth and differentiation. *J Biol Chem* **279:** 54510-54517

Rengby O, Johansson L, Carlson LA, Serini E, Vlamis-Gardikas A, Karsnas P, Arner ES (2004) Assessment of production conditions for efficient use of Escherichia coli in high-yield heterologous recombinant selenoprotein synthesis. *Appl Environ Microbiol* **70:** 5159-5167

Vedadi M, Niesen FH, Allali-Hassani A, Fedorov OY, Finerty PJ, Jr., Wasney GA, Yeung R, Arrowsmith C, Ball LJ, Berglund H, Hui R, Marsden BD, Nordlund P, Sundstrom M, Weigelt J, Edwards AM (2006) Chemical screening methods to identify ligands that promote protein stability, protein crystallization, and structure determination. *Proc Natl Acad Sci U S A* **103:** 15835-15840

Winn MD, Ballard CC, Cowtan KD, Dodson EJ, Emsley P, Evans PR, Keegan RM, Krissinel EB, Leslie AG, McCoy A, McNicholas SJ, Murshudov GN, Pannu NS, Potterton EA, Powell HR, Read RJ, Vagin A, Wilson KS (2011) Overview of the CCP4 suite and current developments. *Acta crystallographica Section D, Biological crystallography* **67:** 235-242

Xia L, Nordman T, Olsson JM, Damdimopoulos A, Bjorkhem-Bergman L, Nalvarte I, Eriksson LC, Arner ES, Spyrou G, Bjornstedt M (2003) The mammalian cytosolic selenoenzyme thioredoxin reductase reduces ubiquinone. A novel mechanism for defense against oxidative stress. *J Biol Chem* **278:** 2141-2146

Xu J, Arner ES (2012) Pyrroloquinoline quinone modulates the kinetic parameters of the mammalian selenoprotein thioredoxin reductase 1 and is an inhibitor of glutathione reductase. *Biochem Pharmacol* **83:** 815-820

Zhong L, Arner ES, Holmgren A (2000) Structure and mechanism of mammalian thioredoxin reductase: the active site is a redox-active selenolthiol/selenenylsulfide formed from the conserved cysteine-selenocysteine sequence. *Proc Natl Acad Sci U S A* **97:** 5854-5859

Scheme S1.

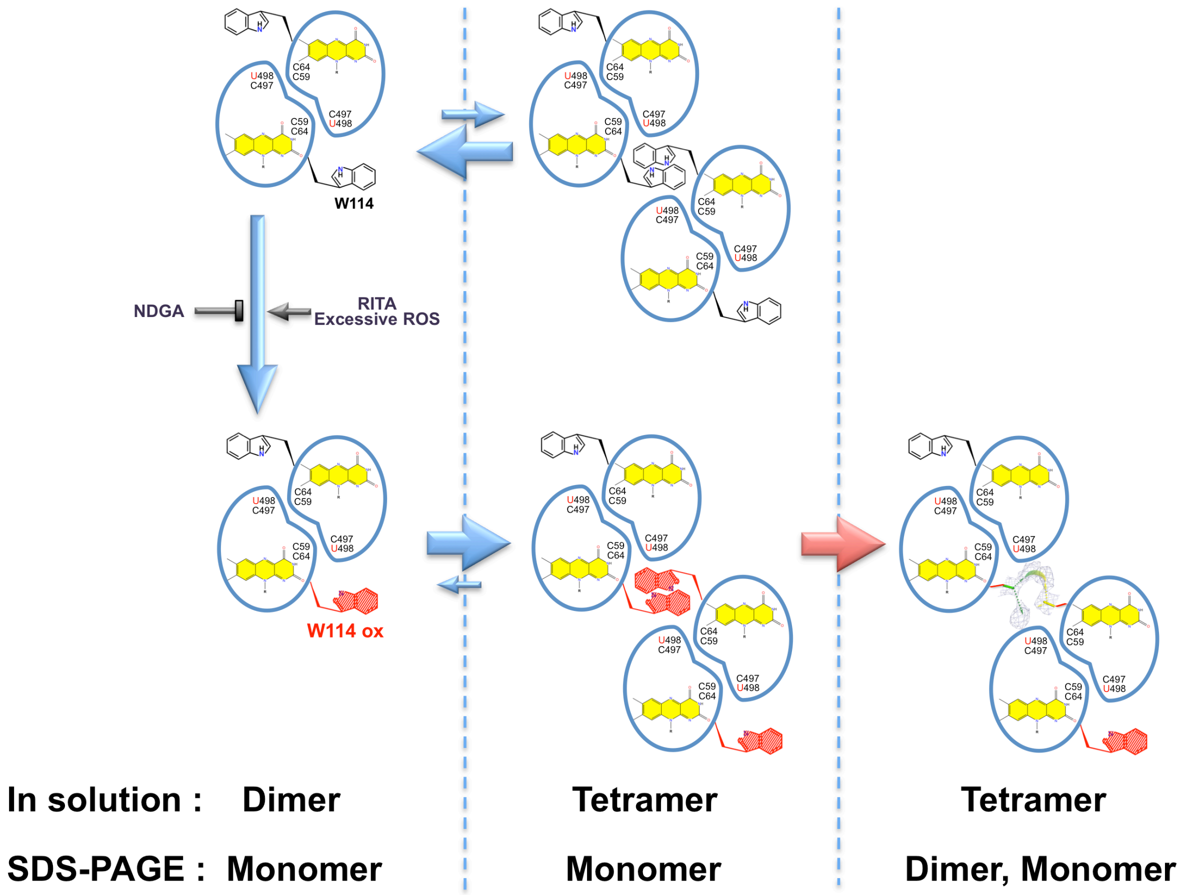

Scheme S1. Dimers and oligomers of TrxR1 as modulated by the Trp114 residue. The different forms of TrxR1 characterized in this study are here schematically summarized. The native form of TrxR1 is mainly present as a dimer, with its two subunits non-covalently associated in a head-to-tail configuration and having the Trp114 residue fully surface-exposed, although a minor fraction of the enzyme species can be seen as non-covalently associated into a tetramer in solution (top of the figure, equilibrium shifted towards the dimeric form). Upon oxidation, as seen either *in vitro* or in cells treated with RITA but prevented by NDGA, the Trp114 residue easily becomes oxidized, which shifts equilibrium towards the tetramer (lower part of the figure) and can subsequently lead to covalent cross-linking between two modified Trp114 residues (lower right, crosslink indicated with a cartoon of the electron density found in the tetramer crystal structure). The resulting forms of TrxR1 detected in solution or in reducing denaturing SDS-PAGE are indicated below the scheme.

Supplementary Figure S1

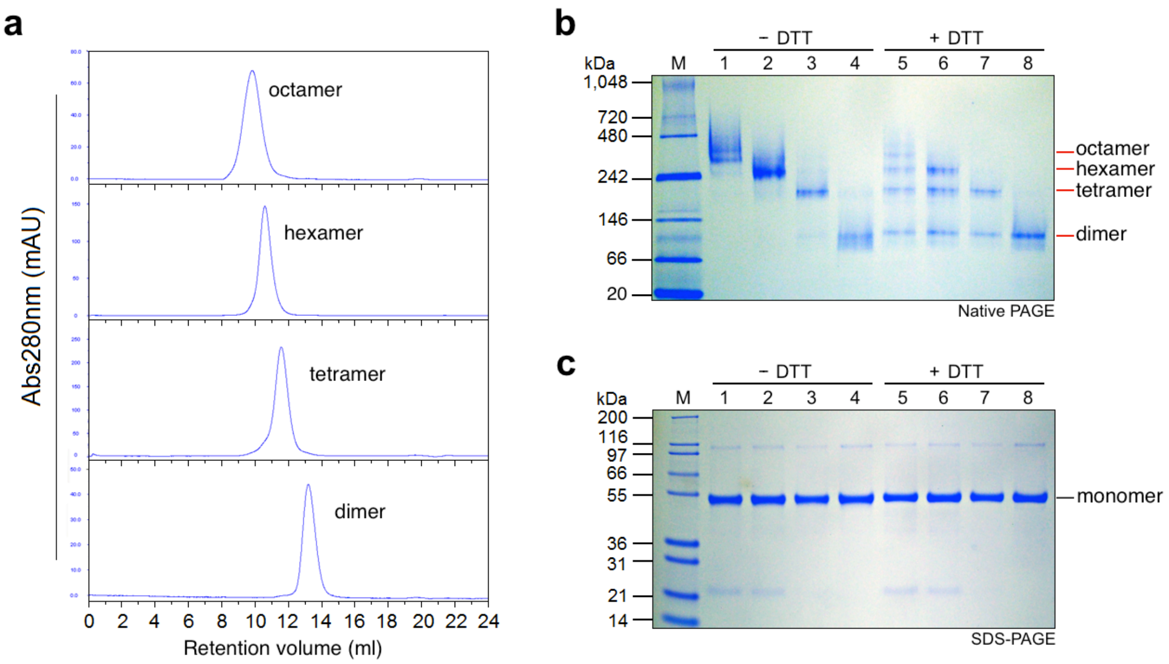

Supplementary Figure S1. Purification of various oligomers of recombinant TrxR1.
(**a**) As purified from 2',5'-ADP Sepharose^{TM} affinity chromatography, recombinant rat TrxR1 oligomeric species were further separated according to their differences of molecular weight (MW) over a Superdex^{TM} G-200 column**,** such as the octamer (MW ≈ 440 kDa), the hexamer (MW ≈ 330 kDa), the tetramer (MW ≈ 220 kDa), and the classical dimeric enzyme (MW ≈ 110 kDa). In solution no monomer of TrxR1 is found. (**b, c**) Both native PAGE and SDS-PAGE analyses of the oligomeric TrxR1 species were performed under either reducing (+DTT) or non-reducing (-DTT) conditions, as indicated. In both the **b** and **c panels**, lanes 1 and 5 represent the TrxR1 octamer; lanes 2 and 6, the TrxR1 hexamer; lanes 3 and 7, the TrxR1 tetramer; lanes 4 and 8, the TrxR1 dimer and lane M, the M12 protein standards (Life Technologies, USA) with sizes indicated in kDa.

Supplementary Figure S2

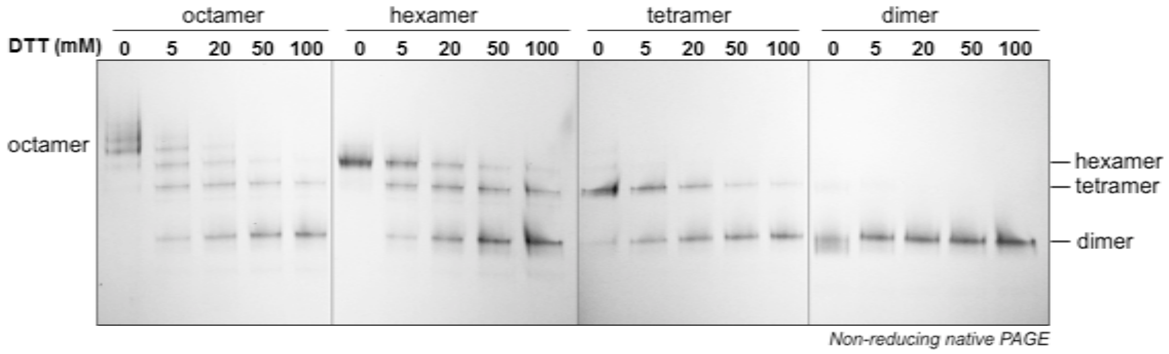

Supplementary Figure S2. TrxR1 oligomers are sensitive towards DTT treatment. Various TrxR1 oligomers together with the regular dimer were used to study the DTT-resistance. After incubation with DTT (5 to 100 mM) for 2 h at room temperature, the samples were mixed with 4 × NativePAGE™ Sample Buffer and then load onto non-reducing NativePAGE™ Novex® 4–16% Bis-Tris gels (Life Technologies, USA). The results show that dissociation of TrxR1 from larger oligomers to smaller oligomers, and finally to dimers, is concentration-dependent and occurs in a step-wise manner.

Supplementary Figure S3

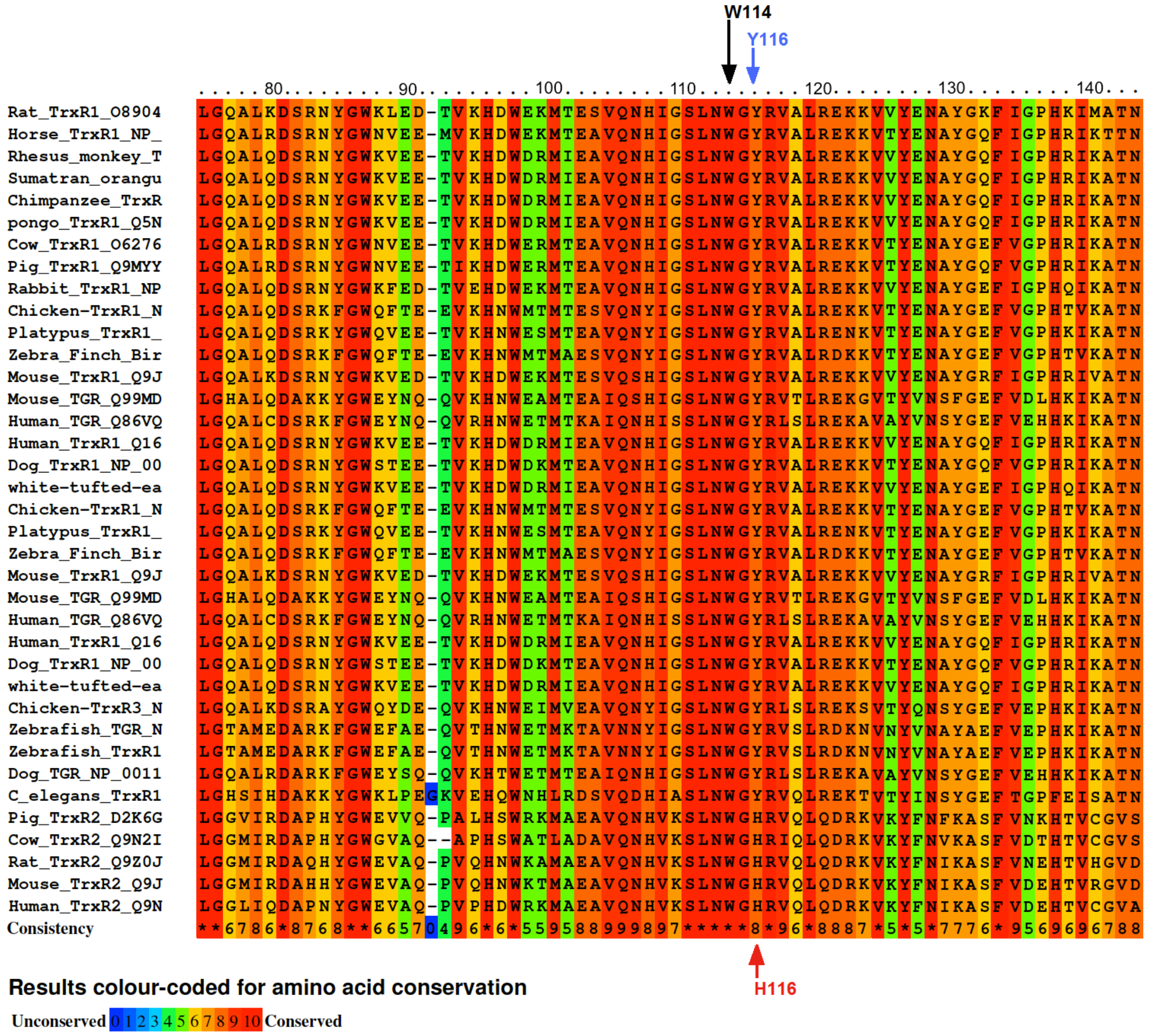

Supplementary Figure S3. Partial sequence alignment of animal TrxRs. The TrxRs used for the sequence alignment were as follows: rat TrxR1 (Swiss-Prot accession no.O89049), mouse TrxR1 (no.Q9JMH6), human TrxR1 (no.Q16881), pongo TrxR1 (no.Q5NVA2), pig TrxR1 (no.Q9MYY8), bovine TrxR1 (no.O62768), horse TrxR1 (NP_001131081; UPI000185554C), dog TrxR1 (NP_001116145; UPI00016DBCE0), rabbit TrxR1 (NP_001243891; UPI000252888A), Rhesus monkey TrxR1 (NP_001243204; UPI000246789D), white-tufted-ear marmoset TrxR (NP_001243348; UPI00024A9A3E), chimpanzee TrxR1 (NP_001116141; UPI00016DBCDF), platypus TrxR1 (no.F6RSS8), chicken TrxR1 (NP_001025933; UPI00015294EF), zebra finch bird TrxR1 (NP_001257893; UPI00027A7715), zebrafish TrxR1 (no.A8WGN7), C. *elegans* TrxR1 (no.Q17745), rat TrxR2 (no.Q9Z0J5), mouse TrxR2 (no.Q9JLT4), human TrxR2 (no.Q9NNW7), pig TrxR2 (no.D2K6G0), bovine TrxR2 (no.Q9N2I8), mouse TGR (no.Q99MD6), human TGR (no.Q86VQ6), dog TGR (NP_001116250.1; UPI000170CD7D), chicken TGR (NP_001116249; UPI000170CD7C), and zebrafish TGR (Q7T2CB). Fully conserved amino acid residues are highlighted in red, such as His^{96}, Trp^{98}, Gln^{106}, His^{108}, Ser^{111}, Leu^{112}, Asn^{113}, Trp^{114}, Gly^{115}, Arg^{117}, Leu^{120} and Val^{125}. Among those amino acid residues found in all organisms, Trp^{114} (marked by a black arrow) is clearly highly conserved. The red arrow indicates His^{116} that is found in mitochondrial TrxR2 instead of Tyr^{116} present in cytosolic TrxR1 or testis-specific TGR (blue arrow).

Supplementary Figure S4

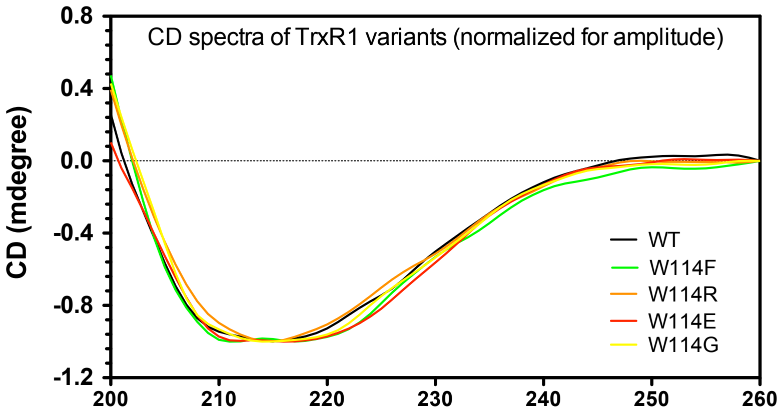

TrxR1 variant	alpha helix	beta sheet	coil	K2d NRMSD
WT (3EA0 crystal)	***0.32***	***0.25***	***0.43***	***n.a.***
WT	0.39	0.20	0.41	0.119
W114F	0.37	0.22	0.41	0.128
W114R	0.33	0.24	0.43	0.140
W114G	0.48	0.21	0.32	0.142
W114E	0.62	0.06	0.31	>0.2

Supplementary Figure S4. Circular dichroism (CD) spectrum analysis. Circular dichroism spectrum analysis was carried out with a 1 cm quartz cuvette at 20 °C using a Jasco™ J-810 Spectropolarimeter with Jasco™ PTC-423S single position Peltier thermostated cell holder (Jasco, Japan). Each sample (800 µl total volume) contained 100nM TrxR1 variants in 1mM Tris-HCl buffer containing 40 nM EDTA (pH 7.5). The CD spectra were scanned from 195 nm to 260 nm with a step size of 1 nm. The buffer spectrum was subtracted from the sample spectra. Secondary structure calculations were subsequently done using the DICHROWEB (http://dichroweb.cryst.bbk.ac.uk/) online tool based on the K2d method, with the results shown in the Table below the graphs. For comparison, the top row of the table lists the secondary structure parameters of our previously determined crystal structure of the wild-type dimeric enzyme (PDB entry 3EA0). All variants analyzed here except W114E displayed good agreement with the secondary structure found in the wildtype dimeric crystal structure, although all displayed normalized root mean square deviation (NRMSD) values above 0.1, thus suggesting some discrepancy between the predicted secondary structures and calculated values. This discrepancy was most pronounced for the W114E variant, suggesting that the calculated secondary structure elements for this variant were less reliable than for the other proteins. n.a.=not applicable

Supplementary Figure S5

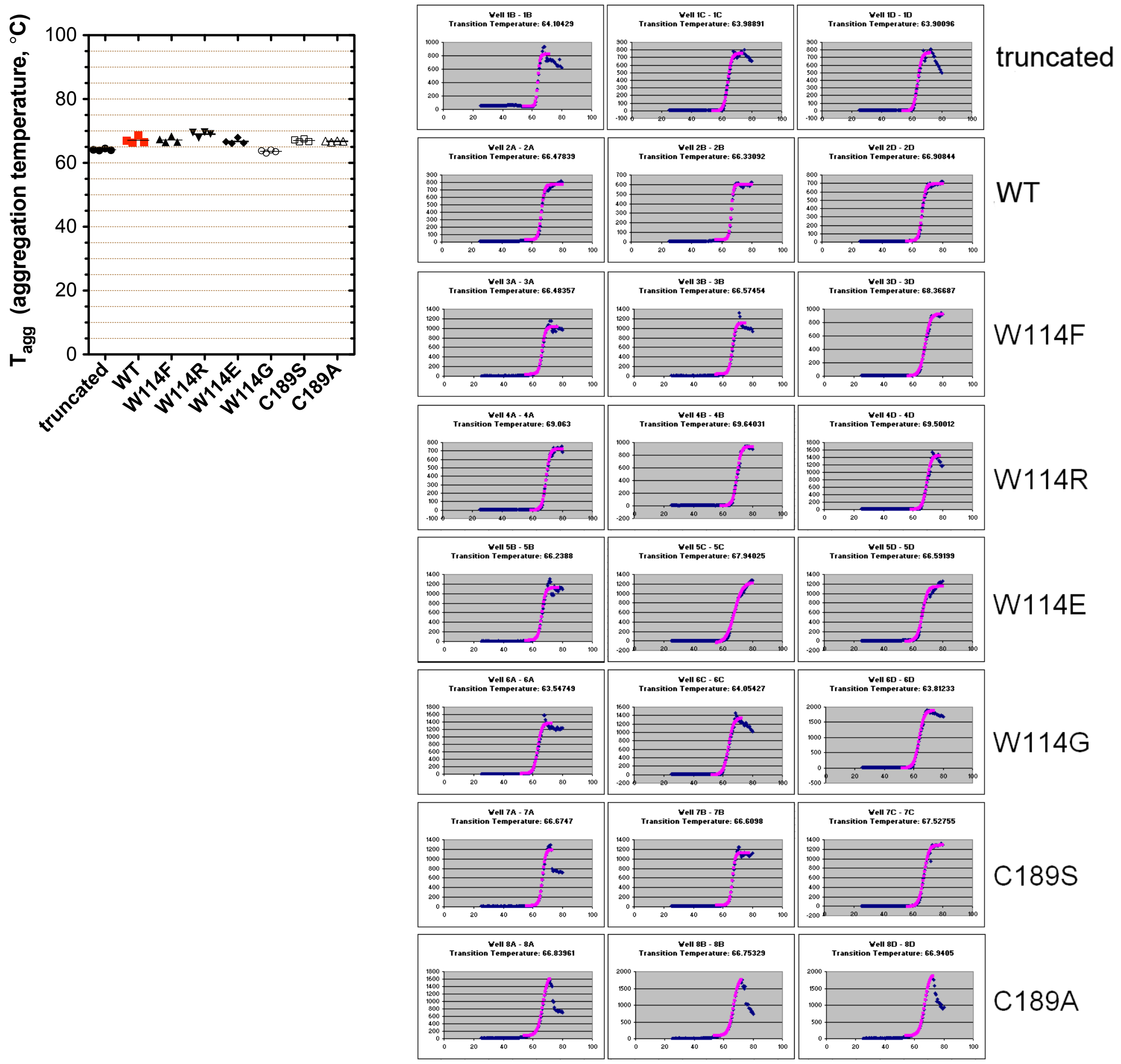

Supplementary Figure S5. Thermostability analysis of TrxR1 variants. Thermostability of TrxR1 variants was measured in parallel by applying a light-scattering-based methodology with a multi-well format StarGazer-384™ instrument (Harbinger Biotechnology and Engineering Corp., Toronto, Canada). TrxR1 samples (4 µM; made by using 30µl of 6.8 µM enzyme stock solution plus 20µl TE buffer pH 7.5, adjusted to a total volume of 50µl) in flat-bottomed 384-well black plates were subjected to the gradually increased temperature from 25 °C to 80 °C at 1°C per min. Mineral oil (40 µl) (M1180, Sigma, USA) was added to the wells to protect sample evaporation. Images of scattered light were taken every 30 s to monitor protein aggregation. The plots showing light intensity vs. temperature were plotted as arbitrary numbers. Intensities were plotted against temperature for each sample well and the transition curves for all samples were fitted with the Boltzmann equation. Finally the thermostability of the protein sample was further evaluated by a T_{agg} parameter (aggregation temperature), which is the temperature value corresponding to the mid-point intensity value (50%) of the melting (or aggregation) curve. Similar T_{agg} indicate similar thermostabilities of the different TrxR1 variants analyzed in the present study.

Supplementary Figure S6

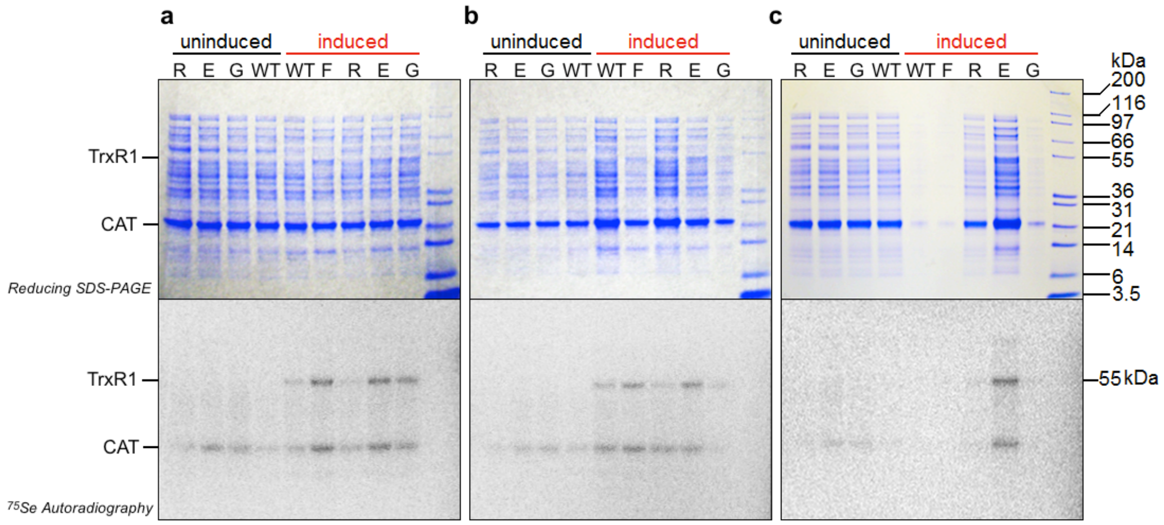

Supplementary Figure S6. ^{75}Se autoradiography analysis of TrxR1 Trp114 mutants. Selenium content i.e. selenocysteine (Sec) content in recombinant rat TrxR1 was evaluated by using ^{75}Se autoradiography. This experiment was performed according to the method described in the text. Radioactive [^{75}Se]selenite (1 µCi/ml) was added to the growth medium before the addition of IPTG to 0.5 mM. The expression of Trp^{114} mutants was IPTG-induced at 24 °C for 4 h and then the soluble supernatants from cell lysates were extracted for measuring TrxR activity, and for performing SDS-PAGE and autoradiography. The upper panels show the Coomassie-stained reducing SDS gels and the bottom panels show the filmless autoradiography. M12 protein standard (Life Technologies, USA) was used as a relative MW indicator. IPTG-induced samples with either the same total protein amount **(a)**, or the same total DTNB reductase activities **(b)**, or the same total Trx-linked insulin reduction activities **(c)** were loaded onto separate reducing SDS-PAGE gels. W114E showed a strong radioactive signal when compared to w.t. TrxR1 and its variants, especially for the gel loaded on the basis of equivalent total activity in the insulin assay. These results indicate that the W114E mutant had high Sec incorporation at its C-terminus, but that the significant loss of TrxR1 function is mainly due to the W114E substitution. Seen as a band in both the protein lysate and the selenium labeling is also the chloramphenicol acetyl transferase (CAT), which is a product of the pSUABC plasmid used for efficient Sec incorporation.

Supplementary Figure S7

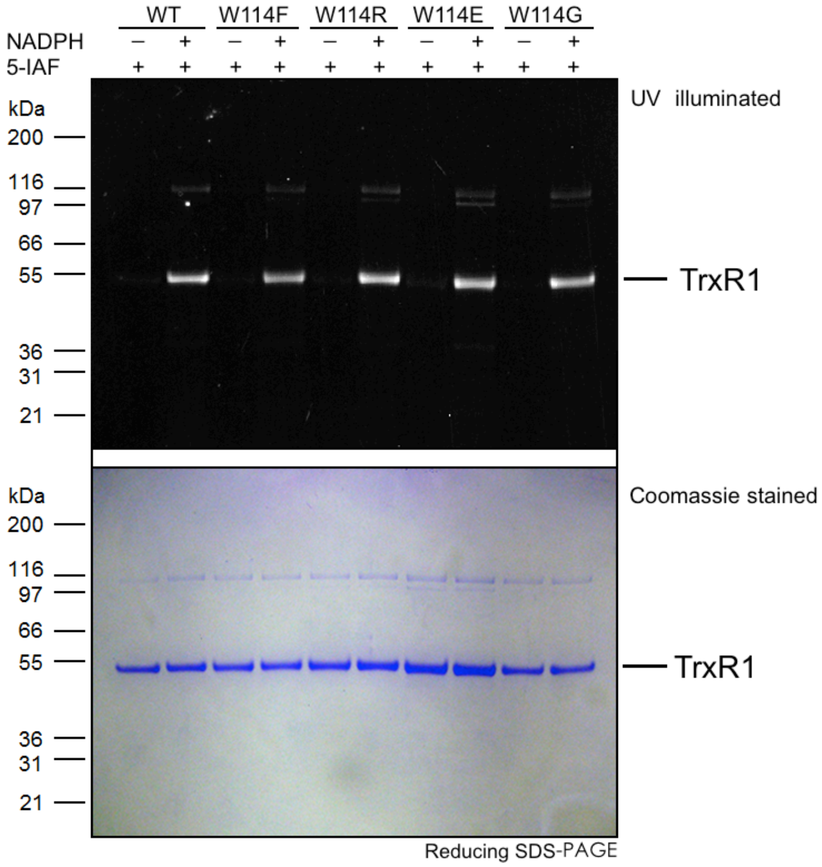

Supplementary Figure S7. 5-IAF labeling. Different TrxR1 variants (500 nM in 50 mM TE buffer, pH 8.5) were reduced with 1 mM NADPH for 15 min at room temperature, followed by addition of 1 mM 5-Iodoacetamidofluorescein (5-IAF, dissolved in DMSO) (Life Technologies, USA) for 30 min darkly at the room temperature. The fluorescent 5-IAF labeled proteins were desalted immediately by using NAP^{TM}-5 columns and eluted using TE buffer (pH 8.5). The labeled products were mixed with 4 × Loading buffer (Life Technologies, USA), denatured at 70 °C for 10 min and further analyzed by the reducing SDS-PAGE with UV illumination (Upper Panel) and Coomassie staining (Bottom Panel). The result shows that fluorescent 5-IAF labels NADPH-reduced TrxR1 samples, but not oxidized TrxR1, indicating that the C-terminal selenolthiol of all the TrxR1 W114 mutants, which is the target of 5-IAF under these conditions, is functional and reduced by NADPH through the normal reductive half-reaction of the enzyme.

Supplementary Figure S8

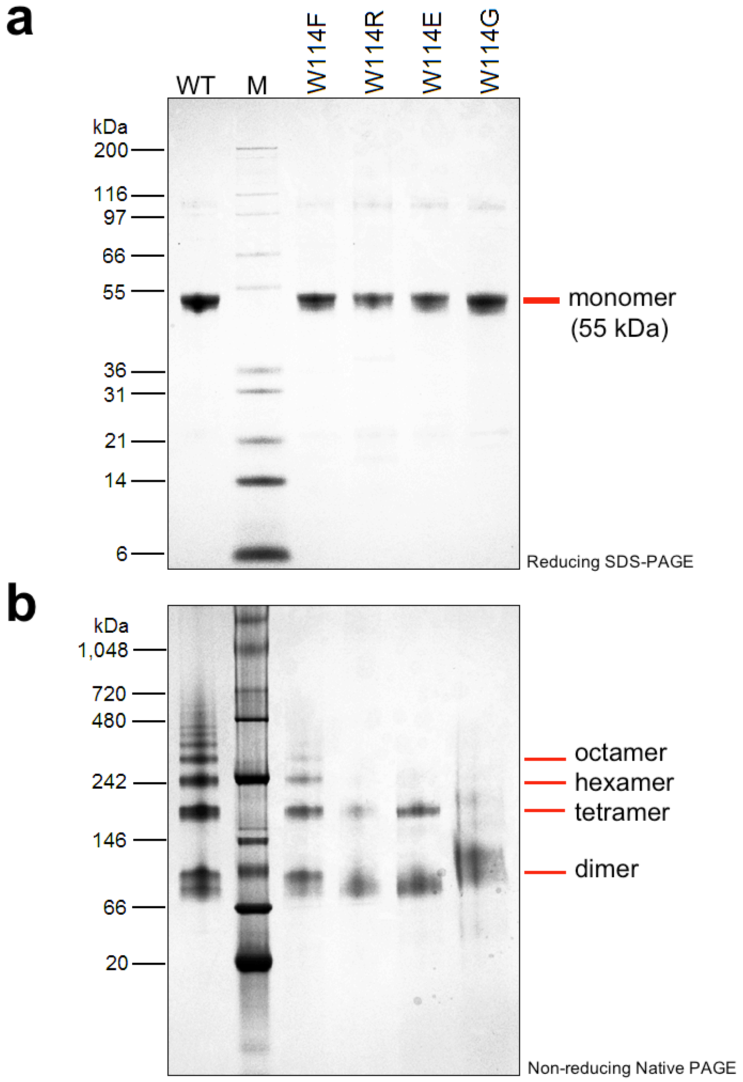

Supplementary Figure S8. PAGE analyses of Trp114 mutants of rat TrxR1. After 0.5 mM IPTG induction at 24 °C for 24 h, four Trp114 mutants of rat TrxR1 were expressed in *E. coli* BL21 (DE3) gor$^-$ host strain and further analyzed by reducing SDS-PAGE (**a**) and non-reducing native PAGE (**b**) In (**a**), the 55 kDa band represents the size of the TrxR1 subunit polypeptide. In (**b**), wild-type rat TrxR1 exhibited clear aggregation behaviour and formed non-covalently linked oligomers in addition to the dimer. Replacement of Trp114 with Arg, Glu or Gly significantly lowered the propensity to form oligomers as in the analyses on non-reducing native PAGE. When Trp was replaced by the aromatic residue Phe, the oligomerization tendency of the enzyme was intermediate.

Supplementary Figure S9

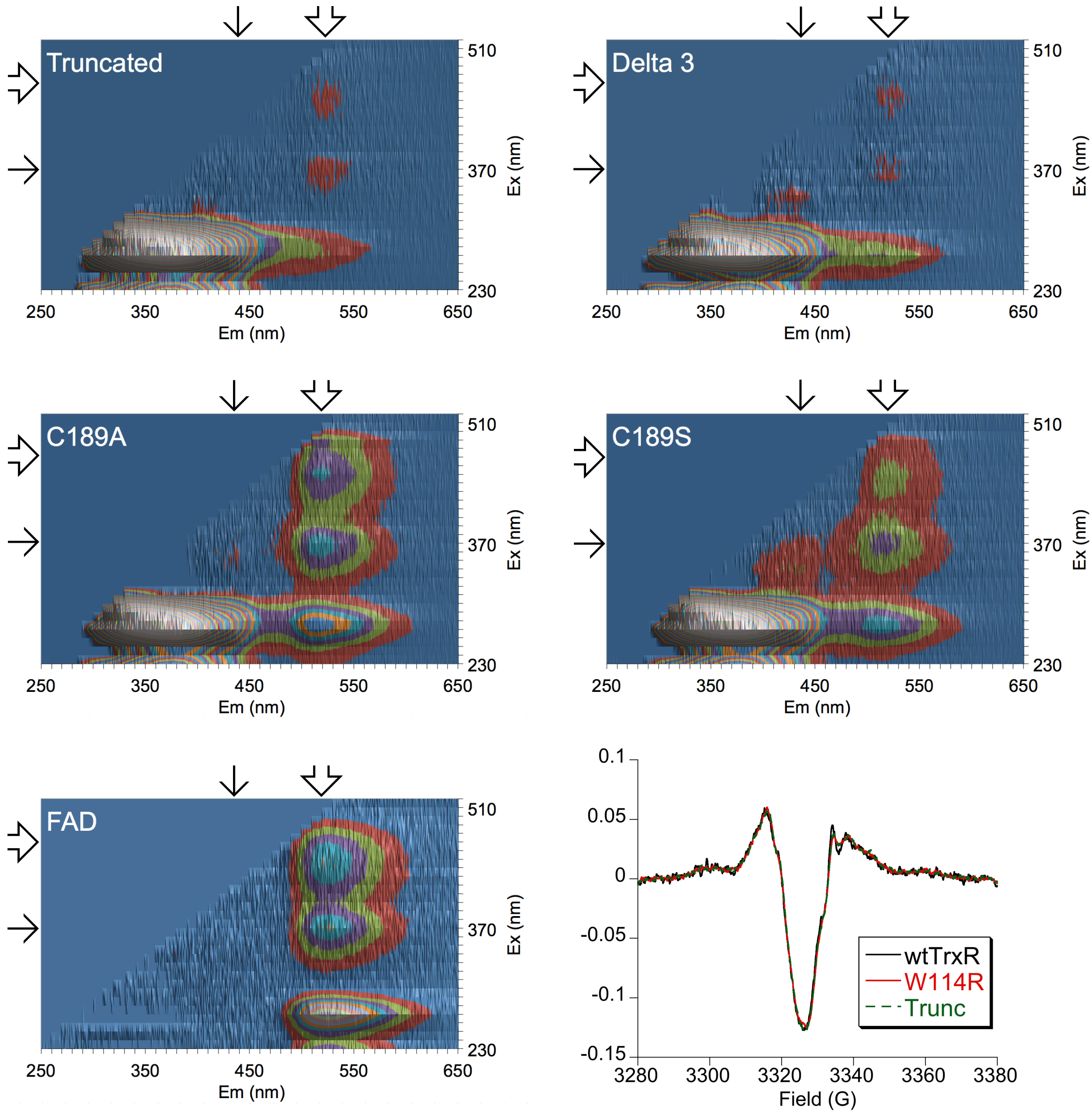

Supplementary Figure S9. 3D fluorescence excitation emission spectra of various TrxR1 variants and free FAD in solution, as well as 2nd harmonic of EPR X-band spectra. The indicated TrxR1 variants (see on-line methods for full descriptions) were dissolved in TE buffer (pH 7.5) and protein concentrations were adjusted to 6.8 µM based on FAD absorbance at 463 nm. In 200 µl (total volume) the TrxR1 samples (or free FAD at a concentration of 2 µM) were loaded into a flat-bottomed 96-well black plate (Thermo Fisher Scientific, USA) for fluorescence measurements using the EnSpire™ Fluorescence Analyzer (Perkin Elmer, USA). The emission spectrum ranging from 250 nm to 650 nm was obtained by exiting the samples first at the excitation wavelength of 230 nm and subsequently the excitation wavelength was increased by 10-nm intervals up to 510 nm. The data obtained for all excitation wavelengths were subsequently used to plot the fluorescence emission spectra in 3D format, with ‘X-axis’ as the excitation wavelength (Ex), ‘Y-axis’ as the emission wavelength (Em), and the ‘Z-axis’ as fluorescence intensity. The TE buffer 3D spectrum was subtracted from all samples. The lower right figure displays the 2nd harmonic of the EPR X-band spectra of wtTrxR, W114R, and Truncated (Sec-minus) TrxR1 variants, where all enzymes were reduced with NADPH to the EH4 state and the EPR signals normalized to the same intensity before the 2nd harmonic was generated so that spectral features could be directly compared.

Supplementary Figure S10

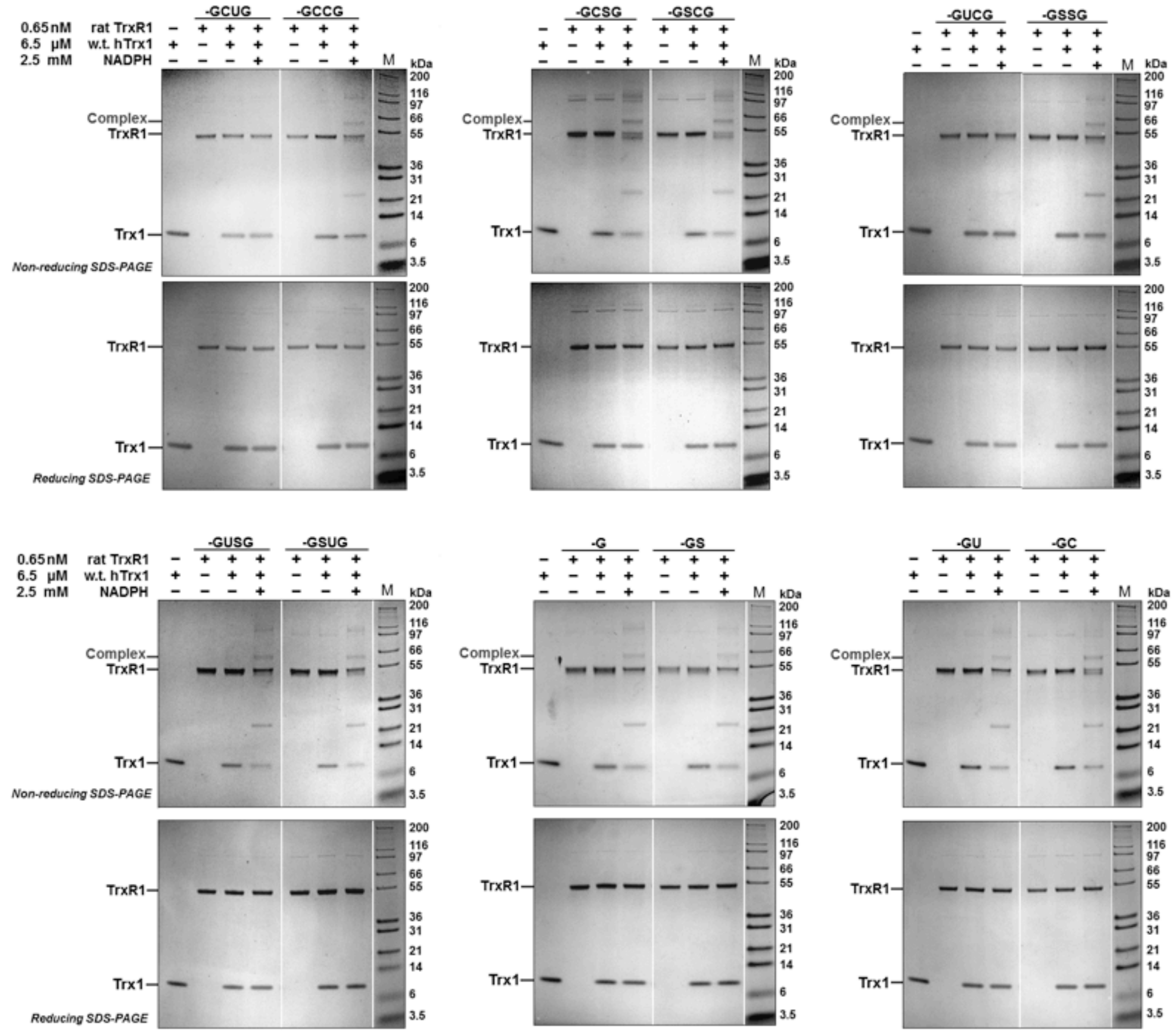

Supplementary Figure S10. Variant TrxR1-Trx1 complexes formed under native conditions. In this experiment, wild type TrxR1 or several variants (C-terminal sequences indicated on top of each gel) were used to probe the formation of disulfide-linked complexes with wild-type human Trx1 with or without the presence of NADPH. In brief, the TrxR1 variants were incubated with human Trx1 in the presence or absence of 2.5 mM NADPH at room temperature for 24 h, whereupon complex formation was analyzed on SDS-PAGE without ("Non-reducing SDS-PAGE") or with ("Reducing SDS-PAGE") addition of 20 mM DTT. All samples were before the SDS-PAGE analyses mixed with 4 × LDS loading solution (Life Technologies, USA) and denatured at 70 °C for 10 min. Note that several TrxR1 variants formed disulfide-linked complexes with Trx1 under these conditions, as illustrated by the absence of a complex in the reducing SDS-PAGE that was seen in the non-reducing SDS-PAGE analyses (indicated in the panels as "Complex").

Supplementary Tables

Table S1. Mass spectrometry analysis of protein bands immunoprecipitated with TrxR1 specific antibodies after treatment of HCT116 cells with RITA. HCT116 cells were treated with 1 μM RITA for 8 h, following which protein in 540 μl eluate was pulled down from cell extracts using immunoprecipitation with anti-TrxR1 antibodies and analyzed on reducing SDS-PAGE, whereupon bands at 52 kDa (band 1), 55 kDa (band 2), 60 kDa (band 3) and 110 kDa (band 4), visualized by Coomassie staining (see **Fig. 1a**). These were subjected to in-gel tryptic digestion and analyses by mass spectrometry using MALDI, which confirmed the presence of TrxR1 in all samples and showed excellent peptide coverage for the applicable peptide mass range (700 – 2,200 Da). The peptide containing the Trp114 residue could not be found in band 4 and was present in several oxidation states in the other bands. See main text for further details.

Residue number [1]	Sequence	Theoretical peptide mass [2] (m/z)	Modification /mass shift [3]	Matching masses (m/z) as detect in MALDI [4]			
				band 4 (110kDa) [5]	band 3 (60kDa) [5]	band 2 (55kDa) [5]	band 1 (52kDa) [5]
30-36	-(K)EAAQYGK(K)-	766.3730		766.3555	766.3539	766.3625	766.3606
38-52	-(K)VMVLDFVTPTPLGTR(W)-	1645.8982		1645.8829	1645.8703	1645.8606	1645.8632
38-52	-(K)VMVLDFVTPTPLGTR(W)-	1661.8931	1 ox M	1661.8705	1661.8587	1661.8471	1661.8521
53-67	-(R)WGLGGTCVNVGCIPK(K)-	1616.7803		1617.7788	1617.7589	1617.7486	1617.7507
69-84	-(K)LMHQAALLGQALQDSR(N)-	1751.9221		1749.8641	1749.8438	1749.8379	1749.8370
90-100	-(K)VEETVKHDWDR(M)-	1413.6757		1413.6808	1413.6715	1413.6483	1413.6620
101-117	-(R)MIEAVQNHIGSLNW[114]GYR(V)-	1987.9807		------[6]	**1987.9812**	**1987.9663**	**1987.9716**
101-117	-(R)MIEAVQNHIGSLN**W**[114]GYR(V)-	2003.9756	1 ox M or 1 ox W	------[6]	**2003.9747**	**2003.9597**	**2003.9718**
101-117	-(R)MIEAVQNHIGSLN**W**[114]GYR(V)-	2019.9705	2 ox W	------[6]	------[6]	**2019.9671**	**2019.9772**
124-139	-(K)KVVYENAYGQFIGPHR(I)-	1877.9657		1877.9512	1877.9404	1877.9403	1877.9425
151-156	-(K)IYSAER(F)-	738.3781		738.3681	738.3714	------[6]	738.3833
157-166	-(R)FLIATGERPR(Y)-	1159.6582		1159.6530	1159.6508	1159.6471	1159.6470
236-246	-(K)IGEHMEEHGIK(F)-	1295.6049	1 ox M	1295.6125	1295.5969	1295.5582	1295.5681
250-266	-(R)QFVPIKVEQIEAGTPGR(L)-	1869.0229		1869.0096	1868.9930	1868.9877	1868.9939

[1] Amino acid residues of human TrxR1 are re-numbered according to the rat TrxR1 sequence (also see **Fig. S3**).
[2] Theoretical peptide masses (MH+) were obtained in using the online MS-digest software from UCSF (http://prospector.ucsf.edu/prospector/).
[3] M is Met, methionine residue; W is Trp, tryptophan residue; ox is oxidation; 1 or 2 indicate that the peptides were modified once or twice, respectively.
[4] Detected peptide masses (MH+) in MADLI and ESI-TOF are matching to human cytoplasmic thioredoxin reductase 1 (Swiss-Port accession no. Q16881).
[5] Samples analyzed in MALDI corresponding to the protein bands of these apparent molecular weights as identified in **Fig. 1a**.
[6] Lack of peptide mass number shows that the corresponding mass could not be found in the indicated sample.

Table S2. Data collection and refinement statistics of tetrameric form of rat TrxR1.

**Data Collection**	
Resolution (Å)	70.0-2.4 (2.53-2.40)
Space group	P31 2 1
Cell dimensions a, b, c (Å)	162.99, 162.99, 236.44
g	120º
Number of reflections	
Observed	*532330 (65569)*
Unique	*137497 (18309)*
<I/s>	8.5 (1.7)
Completeness (%)	97.3 (89.6)
R_{merge} (%)	9.4 (67.1)
Number of monomers/asymmetric unit	4
Solvent content in the crystal	69%
B-factor from Wilson plot (Å^2)	46.0
**Refinement Statistics**	
R_{cryst} (%)	16.0
R_{free} (%)	18.7
Number of atoms	
Total	*15786*
Protein	14975
FAD	212
Solvent	*527*
MPD	*72*
B-factor (Å^2)	
Average	*32.6*
Protein main chains	*29.9*
Protein side chains	36.1
FAD	46.7
Solvent	46.7
Rmsd from ideal geometry	
Bond length	*0.019*
Bond angles	1.82
Ramachandran plot (%)	
Residues in most favored regions	96.6
Residues in disallowed regions	*0*

Figures in parentheses correspond to the highest resolution shell

* 5% of reflections have been used for the monitoring of the refinement

Table S3. Mass spectrometry analysis of tryptophan oxidation in TrxR1 tetramer and dimer. The dimer and tetramer bands of TrxR1 were cut from the non-reducing native PAGE gel and followed by in-gel tryptic digestion, subsequently used for mass spectrometry analyses. Both MALDI-TOF and ESI-TOF mass spectra were combined to identify the distinct peptide printing from the TrxR1 tetramer and dimer preparation. Trp114 oxidation was observed in the TrxR1 tetramer but not in the dimer.

Residue number	Sequence	Theoretical Peptide mass [1] (m/z)	Modification /mass shift [2]	Matching masses as detect in MALDI or ESI-TOF (m/z) [3]	
				Tetramer	Dimer
151-156	-(K)VYSAER(F)-	724.3624		------[4]	724.3518
357-364	-(R)LYGGSTVK(C)-	824.4512		------[4]	824.4392
167-174	-(R)YLGIPGDK(E)-	862.4669		------[4]	862.4507
149-156	-(K)EKVYSAER(F)-	981.5000		------[4]	981.5007
299-308	-(R)TIGLETVGVK(I)-	1016.5986		------[4]	1016.5922
227-235	-(R)GFDQDMANK(I)-	1025.4357		------[4]	1025.4203
125-133	-(K)VVYENAYGK(F)-	1042.5204		1042.5025	1042.5051
1-9	MNDSKDAPK(S)-	1064.4565	1 ox M, 1 Acetyl	------[4]	1064.4912
157-166	-(R)FLIATGERPR(Y)-	1159.6582		1159.6600	1159.6602
256-266	-(K)IEQIEAGTPGR(L)-	1170.6113		1170.6146	1170.6128
425-434	-(K)VICNLKDNER(V)-	1260.6365	1 Cam	------[4]	1260.6459
340-351	-(K)LELTPVAIQAGR(L)-	1267.7369		1267.7501	1267.7415
236-246	-(K)IGEHMEEHGIK(F)-	1279.6099		------[4]	1279.6126
236-246	-(K)IGEHMEEHGIK(F)-	1295.6049	1 ox M	1295.6036	1295.5992
69-81	-(K)LMHQAALLGQALK(D)-	1393.7984		------[4]	1393.7783
90-100	-(K)LEDTVKHDWEK(M)-	1399.6852		------[4]	1399.6876
69-81	-(K)LMHQAALLGQALK(D)-	1409.7933	1 ox M	------[4]	1409.7871
68-81	-(K)KLMHQAALLGQALK(D)-	1537.8883	1 ox M	1537.9043	1537.8827
53-67	-(R)WGLGGTCVNVGCIPK(K)-	1617.7876	2 Cam	1617.8041	1617.7989
38-52	-(K)VMVLDFVTPTPLGTR(W)-	1645.8982		1645.9299	1645.9134
38-52	-(K)VMVLDFVTPTPLGTR(W)-	1661.8931	1 ox M	1661.9095	1661.8938
37-52	-(K)KVMVLDFVTPTPLGTR(W)-	1789.9881	1 ox M	1790.0334	1789.9899
250-266	-(R)QFVPTKIEQIEAGTPGR(L)-	1871.0021		1871.0352	1871.0085
10-29	-(K)SYDFDLIIIGGGSGGLAAAK(E)-	1925.0015		------[4]	1925.0332
101-117	-(K)MTESVQNHIGSINW[114]GYR(V)-	1991.9392		------[4]	1991.9513
101-117	-(K)MTESVQNHIGSINW[114]GYR(V)-	1993.9550		1993.9720	------[4]
101-117	-(K)MTESVQNHIGSIN**W**[114]GYR(V)-	2007.9341	1 ox M, or 1 ox W	2007.9531	2007.9324
101-117	-(K)MTESVQNHIGSIN**W**[114]GYR(V)-	2011.9291	1 ox M + 2 ox W - CO	**2011.9000**	------[4]
101-117	-(K)MTESVQNHIGSIN**W**[114]GYR(V)-	2023.9290	1 ox M + 1 ox W, or 2 ox W	**2023.9436**	------[4]
101-117	-(K)MTESVQNHIGSIN**W**[114]GYR(V)-	2039.9240	1 ox M + 2 ox W	**2039.9546**	------[4]
435-457	-(R)VVGFHVLGPNAGEVTQGFAAALK(C)-	2282.2292		2282.2500	2282.2135
273-293	-(K)STNSEETIEDEFNTVLLAVGR(D)-	2324.1252		2324.1300	2324.1149
462-485	-(K)QQLDSTIGIHPVCAEIFTTLSVTK(R)-	2658.3807	1 Cam	2658.3656	2658.3987
316-339	-(K)IPVTDEEQTNVPYIYAIGDILEGK(L)-	2677.3607		2677.4621	2677.3900
394-416	-(K)FGEENIEVYHSFFWPLEWTVPSR(D)-	2869.3620		2869.3710	2869.3193

[1] Theoretical peptide masses (MH+) were obtained in using the online MS-digest software from UCSF (http://prospector.ucsf.edu/prospector/).

[2] Detected peptide masses (MH+) in MADLI and ESI-TOF are matching to rat cytosolic thioredoxin reductase 1 (Swiss-Port accession no. O89049).

[3] M is Met, methionine residue; W is Trp, tryptophan residue; ox is oxidation; Actyl is acetylation; Cam is carbamidomethylation; - CO is one carbon monoxide removed; 1 or 2 indicate that the peptides are modified either once or twice, respectively.

[4] Lack of peptide mass number indicates that the corresponding mass could not be found in the analyzed tetramers or dimeric TrxR1 samples.

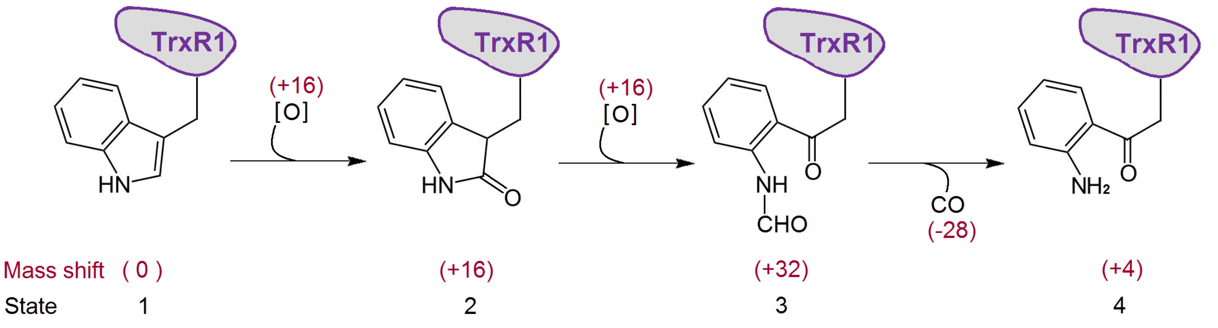

Table S4. Normalization of turnover of TrxR1 variants for selenium content. The turnover numbers of the TrxR1 variants (panel a) were adopted form **Table 1** (main text) to be normalized for their selenium content, determined in duplicate using elemental analysis (results shown as percentages in ***panel a***). Thereafter the selenium content values were used to normalize the turnover numbers as shown in ***panel b***. The selenium contents of the purified TrxR1 variants were determined by elemental analysis at ALS Scandinavia AB, Luleå, Sweden and were calculated as the estimated percentages of the TrxR1 subunits in each TrxR1 variant that contained selenium.

a

Kcat original	w.t.	W114F	W114R	W114E	W114G	C189S
**Se content**	**53.68%**	**56.82%**	**34.79%**	**91.50%**	**56.70%**	**24.81%**
DTNB	2548	1312	1192	326.7	1003	1058
Trx1	1130	595.7	105.9	3	101.9	682.8
Selenite	478.7	270.5	216	104.2	201.7	215.6
9,10-PQ	1718	958.6	844.4	309.3	784.8	634.8
Lipoamide	597.2	338.5	320.4	137.1	227.9	386

b

Kcat normalized	w.t.	W114F	W114R	W114E	W114G	C189S
**Se content**	**100%**	**100%**	**100%**	**100%**	**100%**	**100%**
DTNB	4746.6	2309.0	3426.3	357.0	1769.0	4264.4
Trx1	2105.1	1048.4	304.4	3.3	179.7	2752.1
Selenite	891.8	476.1	620.9	113.9	355.7	869.0
9,10-PQ	3200.4	1687.1	2427.1	338.0	1384.1	2558.6
Lipoamide	1112.5	595.7	921.0	149.8	401.9	1555.8
